# Near infrared emissions from both high efficient quantum cutting (173%) and nearly-pure-color upconversion in NaY(WO_4_)_2_:Er^3+^/Yb^3+^ with thermal management capability for silicon-based solar cells

**DOI:** 10.1038/s41377-023-01365-2

**Published:** 2024-01-16

**Authors:** Duan Gao, Baojiu Chen, Xuezhu Sha, Yuhang Zhang, Xin Chen, Li Wang, Xizhen Zhang, Jinsu Zhang, Yongze Cao, Yichao Wang, Lei Li, Xiangping Li, Sai Xu, Hongquan Yu, Lihong Cheng

**Affiliations:** School of Science, Dalian Maritime University, Dalian, 116026 Liaoning China

**Keywords:** Solar energy and photovoltaic technology, Near-infrared spectroscopy

## Abstract

Raising photoelectric conversion efficiency and enhancing heat management are two critical concerns for silicon-based solar cells. In this work, efficient Yb^3+^ infrared emissions from both quantum cutting and upconversion were demonstrated by adjusting Er^3+^ and Yb^3+^ concentrations, and thermo-manage-applicable temperature sensing based on the luminescence intensity ratio of two super-low thermal quenching levels was discovered in an Er^3+^/Yb^3+^ co-doped tungstate system. The quantum cutting mechanism was clearly decrypted as a two-step energy transfer process from Er^3+^ to Yb^3+^. The two-step energy transfer efficiencies, the radiative and nonradiative transition rates of all interested 4 *f* levels of Er^3+^ in NaY(WO_4_)_2_ were confirmed in the framework of Föster-Dexter theory, Judd-Ofelt theory, and energy gap law, and based on these obtained efficiencies and rates the quantum cutting efficiency was furthermore determined to be as high as 173% in NaY(WO_4_)_2_: 5 mol% Er^3+^/50 mol% Yb^3+^ sample. Strong and nearly pure infrared upconversion emission of Yb^3+^ under 1550 nm excitation was achieved in Er^3+^/Yb^3+^ co-doped NaY(WO_4_)_2_ by adjusting Yb^3+^ doping concentrations. The Yb^3+^ induced infrared upconversion emission enhancement was attributed to the efficient energy transfer ^4^I_11/2_ (Er^3+^) + ^2^F_7/2_ (Yb^3+^) → ^4^I_15/2_ (Er^3+^) + ^2^F_5/2_ (Yb^3+^) and large nonradiative relaxation rate of ^4^I_9/2_. Analysis on the temperature sensing indicated that the NaY(WO_4_)_2_:Er^3+^/Yb^3+^ serves well the solar cells as thermos-managing material. Moreover, it was confirmed that the fluorescence thermal quenching of ^2^H_11/2_/^4^S_3/2_ was caused by the nonradiative relaxation of ^4^S_3/2_. All the obtained results suggest that NaY(WO_4_)_2_:Er^3+^/Yb^3+^ is an excellent material for silicon-based solar cells to improve photoelectric conversion efficiency and thermal management.

## Abstract (web summary)

The Yb^3+^ emissions from both the quantum cutting and nearly-pure infrared upconversion and excellent temperature detection were realized in Er^3+^/Yb^3+^ co-doped NaY(WO_4_)_2_ phosphors.

## Graphical abstract

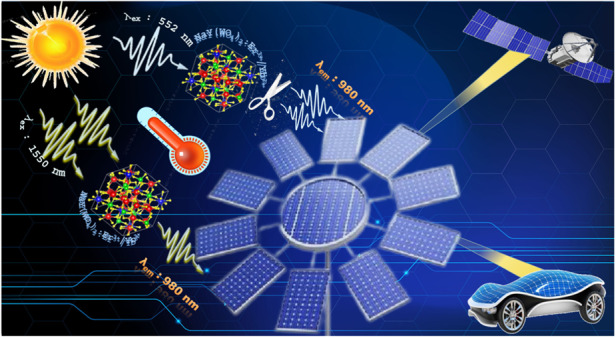

## Introduction

The total amount of widely used fossil fuels on Earth is limited and decreases at an accelerating rate day by day owing to the increase of world population and the expansion of industrial scale^1–4^. Moreover, the continuous use of fossil fuels will result in worldwide energy depletion, serious environmental pollution, and greenhouse gas emissions. Therefore, an urgent task is to find clean and renewable energies to replace fossil fuels^5–7^. Amongst all renewable energies, including wind energy, tidal energy, nuclear energy, hydro-energy, and solar energy, the solar energy is highly favored. Consequently, in recent years, solar cells that can convert light energy into electrical energy have attracted growing interest, and many kinds of solar cells, such as silicon solar cells, dye-sensitized solar cells, cadmium telluride thin film solar cells, perovskite solar cells, quantum dots solar cells, and organic solar cells, have been developed^8–11^. Amongst all of these solar cells, the silicon-based solar cells are the most technically mature and well-commercialized. Usually, it is acceptable in practical applications if the photoelectric conversion efficiency of silicon-based solar cells is larger than 15%. In fact, the efficiency of 15% is just close to the half of its theoretical value of the silicon-based solar cells, and even 1% increase in efficiency is beneficial to the economic profits and promoting applications. Increasing the efficiency through technical improvement for the traditional silicon-based solar cells will greatly increase the economic expenses and lower the cost performance and that is not preferred.

Except for the technical improvement of the traditional solar cell processes, another route to raising the silicon-based solar cell efficiency is to introduce light conversion materials. One of the light conversion materials is capable of converting one photon with energy close to or higher than the two times silicon bandgap energy into two or more photons with energy larger than the silicon bandgap energy. This conversion is widely known as quantum cutting or down-conversion^12–15^. In the silicon-based solar cells, the photocurrent generated by quantum-cut photons can double in comparison with that before quantum cutting, thus further elevating the energy yield of the solar cells. Another type of light conversion materials for silicon-based solar cells is so-called upconversion materials, which can combine two or more infrared photons with single-photon energy lower than silicon bandgap energy to one photon with energy higher than the silicon bandgap energy^16–19^. This upconversion can expand the effective wavelength range of silicon-based solar cells, thus further elevating the energy yield of the solar cells. It should be mentioned that the use of either quantum cutting materials or upconversion materials can increase the electric energy yield of the silicon-based solar cells with no need of changing any performance of the silicon-based solar cells.

The photovoltaic devices must operate when they are exposed to sunlight. Especially, the concentrator solar cells suffer from high-intensity sunlight irradiation^20–23^. The high-intensity sunlight irradiation can result in a high temperature of the solar cells. At the high temperature, the photoelectric conversion efficiency will decrease since the electron and hole recombination efficiency will increase. Therefore, the thermal management for the solar cells cannot be disregarded. The advanced thermal management asks for the temperature measurements. Nowadays, the temperature can be detected via many techniques, for example, thermistor temperature measurement, thermocouple temperature measurement, infrared radiation temperature measurement, fluorescence temperature measurement and so on^24–26^. For the practical application of temperature measurement in solar cells, the technical limit, economic cost, and longevity must be taken into account. Amongst all these temperature measurement routes mentioned above, the fluorescence temperature measurement may be a better choice since the inorganic fluorescence materials can be easily injected into the silicon-based solar cells in a close contact way, and the fluorescence signal for detecting temperature can be read out in a contactless way. In addition, the inorganic fluorescence materials are chemically and physically stable. Therefore, finding a suitable fluorescence-based temperature-measuring material is a key step to advanced thermal management for solar cells.

In recent years, aiming at improving the performance of silicon-based solar cells, both quantum cutting and upconversion materials are widely studied^27–35^. Though the temperature sensing rooting in the spectral measurements is widely reported^36–38^, there are no attempts to use the fluorescence temperature measurement to realize thermal management for solar cells. To our best knowledge, each research paper is concentrated on one issue amongst quantum cutting, upconversion, and temperature sensing. In this work, we attempt to develop a material that integrates quantum cutting, upconversion, and temperature sensing into one together. To this end, the NaY(WO_4_)_2_ is chosen as host for the quantum cutting study of Er^3+^ and Yb^3+^ duo since it has moderate phonon energy, which enables effective nonradiative relaxations from the upper energy level to the lower quantum cutting level (responsible for the first step of quantum cutting) and relatively weak nonradiative transition of the lower quantum cutting level. Moreover, the rare earth ions in NaY(WO_4_)_2_ usually exhibit large transition rates^39–41^. Meanwhile, the WO_4_^2-^ group containing 5 ions can effectively space out the rare earth luminescence centers in NaY(WO_4_)_2_ host and further repress the depopulation effect of the quantum cutting levels via cross-relaxation between rare earth ions and self-generated fluorescence quenching^42^, thus benefiting the quantum cutting emission at high doping concentration. Moreover, Y^3+^ inclusion in NaY(WO_4_)_2_ host is beneficial to the high concentration doping of rare earth luminescence centers. Er^3+^ is chosen as the donor ion since it has uniformly distributed energy level structure in the energy space that may be helpful to the multistep energy transfers from donor to acceptor. Yb^3+^ is chosen as the acceptor ion since it has simplest energy level structure and suitable emission wavelength^43,44^.

In this work, the optimum Er^3+^ concentration was confirmed by taking the visible fluorescence quenching into account, and then the concentration-varied Yb^3+^ were introduced into the Er^3+^ concentration-optimized samples for the quantum cutting investigations. The quantum cutting mechanism was discovered by the optical spectroscopic analyses, and the quantum cutting efficiencies were calculated in assistance of Judd-Ofelt theory, Föster-Dexter theory, energy gap law. The nearly pure color upconversion emission of Yb^3+^ was observed under 1550 nm excitation, and the upconversion mechanism was conducted. Moreover, the temperature sensing based on the fluorescence intensity ratio was studied, and the thermal quenching mechanism of ^4^S_3/2_ green emission was assigned to nonradiative transition. All these results indicate that NaY(WO_4_)_2_:Er^3+^/Yb^3+^ phosphor integrating quantum cutting, upconversion, and temperature sensing in one system is an excellent material especially for the silicon-based concentrating solar cells in which it can be coated on the reflector side surface to realize the light conversions^45^.

## Experimental section

### Sample preparation

Tungstate phosphors NaY_(1-*x*)_Er_*x*_(WO_4_)_2_ (*x* = 0.5, 1.0, 2.0, 5.0, 10.0, 20.0 and 50.0) and NaY_(0.95-*y*)_Er_0.5_Yb_*y*_(WO_4_)_2_ (*y* = 0.5, 1.0, 2.0, 5.0, 10.0, 20.0 and 50.0) were prepared via a high-temperature solid-state reaction method. The raw materials, Y_2_O_3_ (99.99%), Er_2_O_3_ (99.99%), and Yb_2_O_3_ (99.99%) were supplied by Shanghai Second Chemical Reagent Factory (China). Other chemicals including Na_2_CO_3_ and WO_3_ were obtained from Tianjin Reagent Chemicals Co Ltd. (China). All the chemicals were analytical grade and no further purification was carried out.

To prepare the samples, the starting materials were weighed according to the designed stoichiometric ratio. Then, the raw materials were ground in an agate mortar for 30 min to mix them evenly. The well-mixed batch was put into an alumina crucible, and then was placed into an electric furnace. After calcined in air at 1000°C for 4 hours, the sample was obtained when the electric furnace was gradually cooled down to room temperature.

### Sample characterization

The crystal phase structure of the prepared samples was checked by an X-ray powder diffractometer (Shimadzu XRD-6000 equipped with Cu Kα1 radiation resource (λ = 0.15406 nm), in a 2θ range from 20° to 70° at a scanning step of 0.02 °/s. Hitachi F-4600 fluorescent spectrometer equipped with an internal 150 W xenon lamp was used to collect the visible luminescence spectra. The infrared luminescence spectra and the fluorescence lifetimes were measured by Edinburgh FLS1000 spectrofluorometer equipped with a sample holder for powders. The UC emission spectra were measured on a Hamamatsu Vis/IR mini- spectrophotometer C10083MD, and an external 1550 nm fiber output laser was conducted as the excitation source. The diffuse-reflection spectra of the samples were measured by a spectrophotometer UV-3600 (Shimadzu, Japan) equipped with an integrating sphere accessory (Ante, China, 206-23851-91). It should be mentioned that the BaSO_4_ powder provided by the spectrometer manufacturer was used as the reference for measuring the diffuse-reflection spectra. A homemade temperature controlling system DMU-450 with a temperature accuracy of better than 0.5 °C was used to control the sample temperature.

## Results and discussion

### Crystal structure

To identify the crystal structure of the obtained samples, the X-ray diffraction (XRD) patterns for all obtained NaY(WO_4_)_2_ phosphors were measured and are shown in Fig. S1 in the supporting information file. It is seen from Fig. S1 that the diffraction patterns of all synthesized samples are in good agreement with the diffraction pattern reported in the JCPDS card No. 48-0886, thus indicating all prepared samples are tetragonal-phased NaY(WO_4_)_2_ polycrystalline powders.

### Quantum cutting of Er^3+^/Yb^3+^ co-doped NaY(WO_4_)_2_

This section primarily concentrates on the quantum cutting properties of Er^3+^/Yb^3+^ co-doped NaY(WO_4_)_2_ phosphors, including the observation of short wavelength absorption, optimization of the doping concentration, discovery of the quantum cutting mechanisms, and calculations of the quantum cutting efficiencies.

#### Short wavelength absorption of Er^3+^ in NaY(WO_4_)_2_

**Fig. 1:**
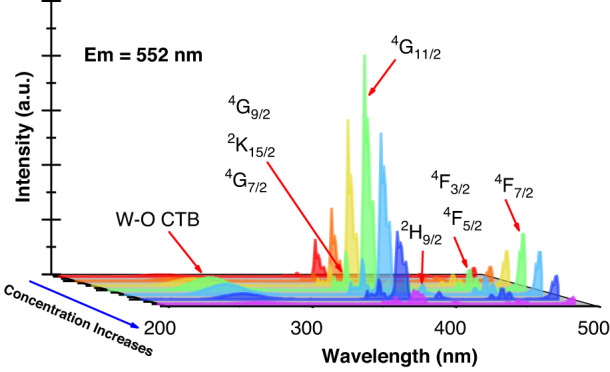
Short wavelength absorption of Er^3+^ in NaY(WO_4_)_2_. Excitation spectra for NaY(WO_4_)_2_:Er^3+^ doped with different Er^3+^ concentrations by monitoring 552 nm emission

To examine the short wavelength absorption of Er^3+^ in NaY(WO_4_)_2_ phosphors, the excitation spectra for the samples with different Er^3+^ concentrations were measured by monitoring 552 nm emission corresponding to ^4^S_3/2_→^4^I_15/2_ transition and are shown in Fig. 1. It is seen that each spectrum contains five narrow absorption peaks and one broad absorption band. As marked in Fig. 1 the five narrow absorption peaks can be assigned to the transitions from ^4^I_15/2_ to ^4^F_7/2_, ^4^F_5/2_/^4^F_3/2_, ^2^H_9/2_, ^4^G_11/2_, and ^4^G_7/2_/^2^K_15/2_/^4^G_9/2_, respectively, but the broad absorption band corresponds to the W^6+^-O^2-^ charge transfer transition. From Fig. 1 it is seen that the NaY(WO_4_)_2_:Er^3+^ exhibits plentiful narrow absorption bands in the range from 350 to 500 nm, and the most intense absorption transition locates at 378 nm corresponding to ^4^I_15/2_→^4^G_11/2_ transition.

#### Er^3+^ concentration optimization

It is known that intense absorption of Er^3+^ for the shorter wavelength will benefit the quantum cutting. The intense absorption asks for a high doping concentration. However, as common sense that the high doping concentration will result in the depopulation of the upper levels via cross relaxations which further depresses the quantum cutting efficiency. Therefore, optimization of the Er^3+^ doping concentration is required.

**Fig. 2:**
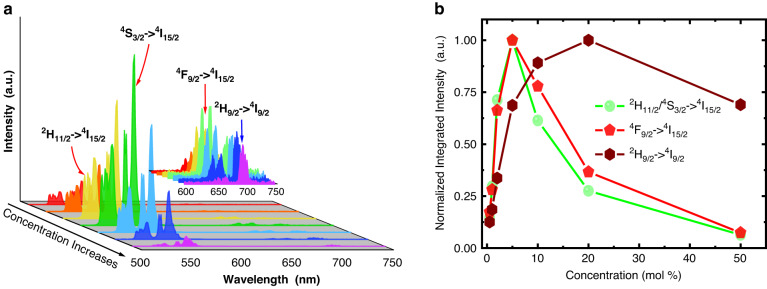
Er^3+^ concentration optimization. **a** Emission spectra of NaY(WO_4_)_2_: *x* mol% Er^3+^ (*x* = 0.5-50) phosphors under 378 nm excitation. **b** Dependences of normalized integrated emission intensities on Er^3+^ concentration

To examine the influence of Er^3+^ concentration on the population of upper metastable state, the emission spectra for all NaY(WO_4_)_2_:Er^3+^ phosphors were measured under 378 nm excitation and are shown in Fig. 2a. It was found that two intense emissions peaking at 530 and 552 nm corresponding to ^2^H_11/2_ → ^4^1_15/2_ and ^4^S_3/2_ → ^4^1_15/2_ transitions are observed. Two very weak emissions peaking at 657 and 700 nm corresponding ^4^F_9/2_ → ^4^1_15/2_ and ^2^H_9/2_ → ^4^I_9/2_ transitions are observed when the spectra ranging from 600 to 750 nm were replotted as in the inset of Fig. 2a. This result also implies that all the levels locating between ^4^G_11/2_ and ^2^H_11/2_ depopulate through cascade nonradiative relaxations since their emissions of these levels are not observed even for the samples with low Er^3+^ concentrations. It is well known that the emission intensity of a level is proportional to its population, therefore, the change of emission intensity reflects the population change. Figure 2b shows the dependences of normalized integrated emission intensities for all observed transitions on the Er^3+^ concentration. From Fig. 2b it can be seen that the emission intensity of the metastable levels ^2^H_11/2_/^4^S_3/2_ reaches its maximum at around 5 mol% of Er^3+^. Therefore, the 5 mol% of Er^3+^ concentration is suggested for designing the Er^3+^/Yb^3+^ co-doped NaY(WO_4_)_2_ quantum cutting phosphors. In addition, the ^4^F_9/2_ as a metastable level may also get involved in the quantum cutting process, but its population under 378 nm excitation is much smaller than that of the levels ^2^H_11/2_/^4^S_3/2_. Therefore, the contribution of level ^4^F_9/2_ to the quantum cutting would be less and can be ignored. Moreover, from Fig. 2b the ^2^H_9/2_ level shows very gentle fluorescence concentration quenching, but its influence on the quantum cutting process is limited since its population is very less in comparison with levels ^2^H_11/2_/^4^S_3/2_.

#### Quantum cutting mechanisms

**Fig. 3:**
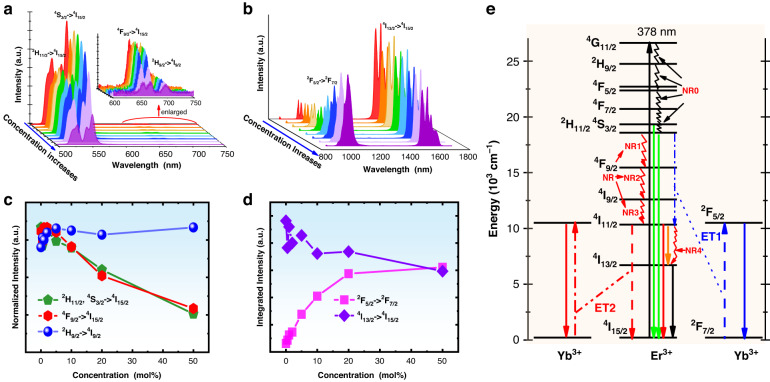
Quantum cutting mechanism. **a** Visible emission spectra, **b** near infrared emission spectra, **c** dependence of normalized visible emission intensity on Yb^3+^ concentration, and **d** dependence of infrared emission intensities of Yb^3+^ and Er^3+^ on Yb^3+^ concentration for NaY(WO_4_)_2_: 5 mol% Er^3+^ / *x* mol% Yb^3+^ phosphors under 378 nm excitation, **e** energy level diagram and quantum cutting routes of Er^3+^/Yb^3+^ doped NaY(WO_4_)_2_ phosphors

In this section, we will explore the quantum cutting mechanisms in Er^3+^/Yb^3+^ co-doped NaY(WO_4_)_2_ phosphors. To this end, the emission spectra of Er^3+^ and Yb^3+^ co-doped NaY(WO_4_)_2_ were measured under 378 nm excitation and are shown in Fig. 3a, b whose wavelength ranging from 500 to 750 nm and from 800 to 1700 nm, respectively. The insert of Fig. 3a displays the enlarged emission spectra in the wavelength range from 600 to 750 nm. It should also be mentioned that there are no emissions observed in the wavelength shorter than 500 nm. This implies that the ^2^H_11/2_/^4^S_3/2_ levels are populated mainly via cascade nonradiative relaxations from ^4^G_11/2_. This also means that the quantum cutting for the levels lying between ^4^G_11/2_ and ^2^H_11/2_ will not happen since these levels were not effectively populated.

From Fig. 3a it can be seen that the samples with varied Yb^3+^ concentrations exhibit four emissions as observed in the Er^3+^ single-doped samples. Figure 3c, d show the dependences of the three visible emission and two NIR emission intensities on the Yb^3+^ concentration, respectively. It is found that though the Er^3+^ concentration is fixed, the emission intensity of ^2^H_11/2_/^4^S_3/2_→^4^I_15/2_ decreases with increasing Yb^3+^ concentration. This fact means that the Yb^3+^ plays a role of depopulating ^2^H_11/2_/^4^S_3/2_ levels, that is to say an energy transfer from Er^3+^ to Yb^3+^ occurs. Based on the energy matching rule, the energy transfer path can be confirmed as ET1: ^4^S_3/2_(Er^3+^)+^2^F_7/2_(Yb^3+^) →^4^I_11/2_(Er^3+^) + ^2^F_5/2_(Yb^3+^). The large energy mismatch between the energy difference from ^4^S_3/2_ to ^4^I_11/2_ and the energy difference from ^2^F_7/2_ to ^2^F_5/2_ means the phonon-assisted energy transfer is involved, and the ET1 happens by annihilating lattice phonons to maintain the energy conservation. After this energy transfer one Er^3+^ gets into ^4^I_11/2_ and one Yb^3+^ gets into ^2^F_5/2_. Analogically, from the dependence of red emission intensity on the Yb^3+^ concentration, an energy transfer ^4^F_9/2_(Er^3+^)+ ^2^F_7/2_(Yb^3+^)→^4^I_13/2_(Er^3+^)+^2^F_5/2_(Yb^3+^) can be deduced, and this energy transfer process is not drawn in Fig. 3e since its contribution to the quantum cutting is very less owing to the ignorable population of ^4^F_9/2_ of Er^3+^ under 378 nm excitation. From Fig. 3b it can be seen that the emission intensity of ^2^H_9/2_ level is almost not changed with Yb^3+^ concentration, thus indicating that there is no any energy transfer originating from ^2^H_9/2_ level. It should also be stated that the impact of ^2^H_9/2_ level on the quantum cutting will also be ignored since the population of this level is less too.

By inspecting the energy level scheme of Er^3+^ and Yb^3+^ in Fig. 3e, it can be deduced that the infrared emission intensity of Yb^3+^ would increase with the increase of Yb^3+^ concentration as a result of the energy transfer ET1, at the same time, the infrared emission intensity of Er^3+^ (corresponding to ^4^I_13/2_→^4^I_15/2_ transition) should synchronously increase due to the increase of ^4^I_13/2_ level population via a nonradiative relaxation from ^4^I_11/2_ level. From the dependences of integrated emission intensities of ^2^F_5/2_→^2^F_7/2_ (Yb^3+^) and ^4^I_13/2_→^4^I_15/2_ (Er^3+^) transitions on the Yb^3+^ concentration in Fig. 3d, it is found that Yb^3+^ emission intensity does increase with increasing Yb^3+^ concentration as we predicted, but the intensity of ^4^I_13/2_→^4^I_15/2_ (Er^3+^) transition decreases with increasing Yb^3+^ concentration which is against our prediction. The decrease of ^4^I_13/2_→^4^I_15/2_ (Er^3+^) transition implies the decrease of ^4^I_13/2_ population with increasing Yb^3+^ concentration. Owing to large energy mismatch, the energy transfer ^4^I_13/2_ (Er^3+^) + ^2^F_7/2_ (Yb^3+^) → ^4^I_15/2_ (Er^3+^) + ^2^F_5/2_ (Yb^3+^) cannot happen. Therefore, the energy transfer ^4^I_11/2_ (Er^3+^) + ^2^F_7/2_ (Yb^3+^) → ^4^I_15/2_ (Er^3+^) + ^2^F_5/2_ (Yb^3+^) (marked as ET2 in Fig. 3e) would occur indubitably. It should be pointed out that if ET2 does not exist, the intensity of ^4^I_13/2_→^4^I_15/2_ (Er^3+^) transition will increase following the same trend as the Yb^3+^ emission intensity does. This is because that the ^4^I_13/2_ is populated via both radiative transition ^4^I_11/2_ → ^4^I_13/2_ and nonradiative relaxation from ^4^I_11/2_, and the ^4^I_11/2_ is populated via the energy transfer ET1 which evokes the increase of Yb^3+^ emission intensity with increasing Yb^3+^ concentration. It should also be noticed that if the population of ^4^I_11/2_ gained via ET1 process is totally contributed to Yb^3+^ via ET2 process, then with increasing Yb^3+^ concentration the intensity of ^4^I_13/2_→^4^I_15/2_ transition will keep a constant intensity as in the 5 mol% Er^3+^ single-doped sample. If the population of ^4^I_11/2_ gained via ET1 process is partially contributed to Yb^3+^ via ET2 process and partially contributed to ^4^I_13/2_ via radiative transition and nonradiative relaxation from ^4^I_11/2_, then the infrared emission of ^4^I_13/2_→^4^I_15/2_ transition will also show an increasing trend when Yb^3+^ concentration increases. The decrease of infrared emission intensity of ^4^I_13/2_→^4^I_15/2_ transition with increasing Yb^3+^ concentration indicates great enhancement of the energy transfer rate of ET2 process with increasing Yb^3+^ concentration.

Based on the above analyses, the specific photon-splitting routes are illustrated in Fig. 3e. In the first step, the Er^3+^ in the ground state ^4^I_15/2_ is promoted to the excited state ^4^G_11/2_ by absorbing a 378 nm photon, and then relaxes rapidly to ^2^H_11/2_/^4^S_3/2_ states. The Er^3+^ in ^2^H_11/2_/^4^S_3/2_ states could be de-excited via three possible routes, namely radiative transition ^2^H_11/2_/^4^S_3/2_→^4^I_15/2_, nonradiative relaxation from ^4^S_3/2_ to ^4^F_9/2_, and energy transfer ET1 ^4^S_3/2_(Er^3+^)+^2^F_7/2_(Yb^3+^)→^4^I_11/2_ (Er^3+^) + ^2^F_5/2_(Yb^3+^). The radiative transitions generate the green emissions as shown in Fig. 1a, and the nonradiative relaxation results in the population of ^4^F_9/2_ and further induces the very weak red emission as shown in the insert of Fig. 1a. The ET1 process can eventuate simultaneously two effects that infrared emission of Yb^3+^ and population of ^4^I_11/2_ of Er^3+^. The Er^3+^ in ^4^I_11/2_ can be de-excited via radiative transitions ^4^I_11/2_→^4^I_15/2_ and ^4^I_11/2_→^4^I_13/2_, nonradiative relaxation to ^4^I_13/2_, and energy transfer ET2. The radiative transition ^4^I_11/2_→^4^I_15/2_ can generate 980 nm photons. The radiative transition ^4^I_11/2_→^4^I_13/2_ and nonradiative relaxation from ^4^I_11/2_ to ^4^I_13/2_ will result in 1550 nm emission. The ET2 process generate infrared emission of Yb^3+^.

#### Quantum cutting efficiency

This section will focus on quantitatively estimating the quantum cutting efficiency. It should be mentioned that the energy transfer efficiency from Er^3+^ to Yb^3+^ cannot be calculated in the way as done for Tb^3+^/Yb^3+^ co-doped system since the energy transfer mechanisms are different. To calculate the quantum cutting efficiencies, the nonradiative transitions from ^4^I_13/2_ to ^4^I_15/2_ and from ^2^F_5/2_ to ^2^F_7/2_ are ignored, but the nonradiative transitions in the possible cases are considered. The radiative transitions are quantitatively evaluated in the framework of Judd-Ofelt calculation.

##### Energy transfer efficiency for ET1 from Er^3+^ to Yb^3+^

**Fig. 4:**
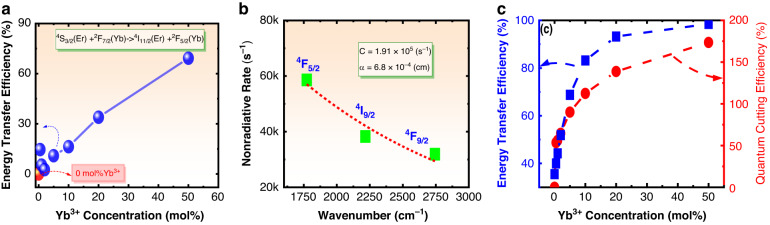
Quantum cutting efficiency. **a** Dependence of energy transfer efficiency of ET1 on Yb^3+^ concentration, **b** Dependence of nonradiative relaxation rate on energy gap: experimental data (solid squares) and fitting curve (dashed), **c** Solid squares show the relationship between the energy transfer efficiency for ET2 and Yb^3+^ concentration; Solid circles show the relationship between the quantum cutting efficiency and Yb^3+^ concentration

The energy transfer efficiency of ET1 can be written as

1 $${{\rm{\eta }}}_{{\rm{ET}}1}=\frac{{I}^{{Er}}-{I}^{{Er}+{Yb}}}{{I}^{{Er}}}$$

where ${I}^{{Er}}$ and ${I}^{{Er}+{Yb}}$ are the luminescence intensities of ^4^S_3/2_→^4^I_15/2_ for 5 mol% Er^3+^ single-doped and 5 mol% Er^3+^/ *x* mol% Yb^3+^ co-doped samples. By taking the integrated luminescence intensity data in Fig. 3b into Eq. (1) the energy transfer efficiencies were calculated, and the dependence of energy transfer efficiency on the Yb^3+^ concentration is shown in Fig. 4a. It can be seen that the energy transfer efficiency dramatically increases with increasing Yb^3+^ concentration, and the energy transfer efficiency can reach 70% when Yb^3+^ concentration is 50 mol%.

##### Radiative transition rates of Er^3+^ in NaY(WO_4_)_2_

From the quantum cutting mechanism shown in Fig. 3e, it can be known that to quantitatively calculate the quantum cutting efficiency, the radiative transition rates between 4 *f* levels of Er^3+^ should be determined. To this end, the Judd-Ofelt calculation for Er^3+^ in NaY(WO_4_)_2_ host should be carried out in advance. In this work, the Judd-Ofelt parameters were obtained via an approach proposed by Zhan Y et al. ^46,47^ using the diffuse reflection spectrum. The specific calculation procedure is presented in supporting information file as the Item <Judd-Ofelt calculation>^48,49^, and the calculation results are listed in Table 1.

**Table 1:** Transition rates, branch ratios, total transition rates, nonradiative transition rates, and radiative lifetimes of Er^3+^ in NaY(WO_4_)_2_ phosphor

Initial level	Final level	Wave-number	Transition rate (S^-1^)		Branch Ratio (%)	Total radiative rate (S^-1^)	Non-radiative rate (S^-1^)	Radiative liifetime (ms)
			ED	MD				
^**4**^**F**_**5/2**_	^**4**^**F**_**7/2**_	1772.32	12.69		0.27	4663.43	57482.33	0.214
	^**2**^**H**_**11/2**_	3065.13	5.08		0.11			
	^**4**^**S**_**3/2**_	3839.87	12.69		0.27			
	^**4**^**F**_**9/2**_	6931.7	314.79		6.75			
	^**4**^**I**_**9/2**_	9675.17	538.19		11.54			
	^**4**^**I**_**11/2**_	11891.64	614.34		13.17			
	^**4**^**I**_**13/2**_	15546.65	2729.01		58.52			
	^**4**^**I**_**15/2**_	22222.22	436.64		9.36			
^**4**^**F**_**7/2**_	^**2**^**H**_**11/2**_	1292.81	5.08		0.06	7928.11	79668.76	0.126
	^**4**^**S**_**3/2**_	2067.55	0.00		0.00			
	^**4**^**F**_**9/2**_	5159.38	50.77		0.64			
	^**4**^**I**_**9/2**_	7902.85	337.64		4.26			
	^**4**^**I**_**11/2**_	10119.32	863.13		10.89			
	^**4**^**I**_**13/2**_	13774.33	2465.00		31.09			
	^**4**^**I**_**15/2**_	20449.9	4206.49		53.06			
^**2**^**H**_**11/2**_	^**4**^**S**_**3/2**_	774.74	0.00		0.00	87282.66	23443.66	0.011
	^**4**^**F**_**9/2**_	3866.57	345.25		0.40			
	^**4**^**I**_**9/2**_	6610.04	926.59		1.06			
	^**4**^**I**_**11/2**_	8826.51	558.50		0.64			
	^**4**^**I**_**13/2**_	12481.52	949.44		1.09			
	^**4**^**I**_**15/2**_	19157.09	84502.88		96.82			
^**4**^**S**_**3/2**_	^**4**^**F**_**9/2**_	3091.83	0.00		0.00	637.20		1.569
	^**4**^**I**_**9/2**_	5835.3	96.47		15.14			
	^**4**^**I**_**11/2**_	8051.77	22.85		3.59			
	^**4**^**I**_**13/2**_	11706.79	147.24		23.11			
	^**4**^**I**_**15/2**_	18382.35	370.64		58.17			
^**4**^**F**_**9/2**_	^**4**^**I**_**9/2**_	2743.47	45.70		0.87	5242.25	29701.50	0.191
	^**4**^**I**_**11/2**_	4959.94	281.79		5.38			
	^**4**^**I**_**13/2**_	8614.95	352.87		6.73			
	^**4**^**I**_**15/2**_	15290.52	4561.89		87.02			
^**4**^**I**_**9/2**_	^**4**^**I**_**11/2**_	2216.47	0.00	4.09	0.57	722.52	42502.27	1.384
	^**4**^**I**_**13/2**_	5871.48	20.31		2.81			
	^**4**^**I**_**15/2**_	12547.05	698.12		96.62			
^**4**^**I**_**11/2**_	^**4**^**I**_**13/2**_	3655.01	45.70	20.30	11.35	581.34	15975.20	1.720
	^**4**^**I**_**15/2**_	10330.58	515.34		88.64			
^**4**^**I**_**13/2**_	^**4**^**I**_**15/2**_	6675.57	165.01	82.83	100	247.82		4.035

##### Nonradiative transition rates of Er^3+^ in NaY(WO_4_)_2_

From Fig. 3e it can be found that the nonradiative transitions are involved in the quantum cutting process, and some of them, for example, the nonradiative transition of ^4^I_11/2_ level, play an important role. Moreover, the cascade nonradiative transitions (NR1, NR2, and NR3) are a pathway for populating ^4^I_11/2_ level. Therefore, to determine the quantum cutting efficiency, the nonradiative transition rates for some levels should be determined in advance.

At a certain temperature, the nonradiative transition rate ${A}_{{nr}}$ of an energy level can be expressed by as follows,

2 $${A}_{{nr}}=C{e}^{-\alpha \triangle E}$$

where *C* is temperature-dependent parameter, and at a not-too-high temperature the parameter *C* can be considered as a constant independent from $\triangle E$; $\alpha$ is a constant in a certain system; $\triangle E$ is the energy gap between the studied energy level and its most adjacent lower energy level. To confirm these parameters *C* and $\alpha$, the fluorescence decays of ^4^F_5/2_, ^4^F_9/2_, and ^4^I_9/2_ levels for 1 mol% Er^3+^ doped NaY(WO_4_)_2_ phosphor were measured and are shown in Fig. S4. It can be seen that all decays display straight lines in the semi-logarithmic coordinate system, thus implying all decays follow the mono-exponential function. Therefore, mono-exponential function was fit to these decays, and the fluorescence lifetimes were confirmed and are marked in the figure. In the sample single-doped with 1 mol% Er^3+^, the energy transfers between Er^3+^ can be ignored since the distance between Er^3+^ is large enough. Therefore, the nonradiative relaxation rate for a level can be expressed as

3 $${A}_{{nr}}=\frac{1}{{\tau }_{{ex}}}-{A}_{r}$$

where ${\tau }_{{ex}}$ is fluorescence lifetime, ${A}_{r}$ is total radiative transition rate as listed in Table 1. By taking the lifetimes obtained from Fig. S4 into Eq. (3), the nonradiative relaxation rates for ^4^F_5/2_, ^4^F_9/2_, and ^4^I_9/2_ levels are confirmed, and the energy gaps from ^4^F_5/2_, ^4^F_9/2_, and ^4^I_9/2_ levels to their own most adjacent lower levels can be obtained from Table 1. The dependence of the nonradiative relaxation rate on the energy gap is shown in Fig. 4b as the solid squares. The data in Fig. 4b were fit to Eq. (2), and the values of the free parameters $C$ and $\alpha$ were confirmed from the fitting process to be 1.9×10^6 ^s^-1^ and 6.8×10^-4 ^cm. Thereby the nonradiative relaxation rates for all level can be derived by taking energy gap value between the studied level and its most adjacent level into Eq. (2). The nonradiative relaxation rates for interested levels of Er^3+^ in NaY(WO_4_)_2_ were calculated and are listed in Table 1 as the second column from right hand. It should be stated that the ^2^H_11/2_ and ^4^S_3/2_ were not involved in the determination of the parameters ${\rm{C}}$ and ${\rm{\alpha }}$ since they are in a thermal equilibrium, and ^4^I_13/2_ was not involved either since the distance between ^4^I_13/2_ and ^4^I_15/2_ is too large.

##### Energy transfer efficiency for ET2 from Er^3+^ to Yb^3+^

The energy transfer rate for ET2 could be calculated based on the Fӧrster-Dexter energy transfer theory, the energy transfer rate ${A}_{{et}}^{D\to A}$ between donors and acceptors can be expressed as follows^49,50^,

4 $${A}_{{et}}^{D\to A}=\frac{3h{c}^{2}{Q}_{A}{Q}_{D}}{4{R}^{6}{\pi }^{3}{n}^{2}}\int \frac{{f}_{D}(E){f}_{A}(E)}{{E}^{2}}{dE}$$

where ${A}_{{et}}^{D\to A}$ is the energy transfer rate for ET2 process from donor Er^3+^ to acceptor Yb^3+^, $h$ is Planck constant, $c$ is the velocity of light in vacuum, ${Q}_{A}$ and ${Q}_{D}$ are the integrated cross-sections for acceptor absorption (Yb^3+^: ^2^F_7/2_→^2^F_5/2_) and donor emission (Er^3+^: ^4^I_11/2_→^4^I_15/2_), ${f}_{A}\left(E\right)$ and ${f}_{D}(E)$ are the normalized spectral line shape functions of acceptor absorption and donor emission, respectively. $n$ is the refractive index of the host NaY(WO_4_)_2_. $E$ is the photon energy, namely h*v*, and $R$ is average distance between Er^3+^ and Yb^3+^ ions in NaY(WO_4_)_2_ and can be calculated based on the following formula^51^,

5 $$R=\frac{0.62}{\root{3}\of{{N}_{A}+{N}_{D}}}$$

where ${N}_{A}$ and ${N}_{D}$ are numbers of Yb^3+^ and Er^3+^ ions in a unit volume, respectively.

The emission cross-section spectrum ${\sigma }_{{em}}^{{Er}}({\rm{\lambda }})$ for ^4^I_11/2_→^4^I_15/2_ transition was calculated based on the Füchtbauer-Ladenburg and is plotted in Fig. S5 as dashed curve. The absorption cross-section ${\sigma }_{{ab}}^{{Yb}}\left({\rm{\lambda }}\right)$ for ^2^F_7/2_→^2^F_5/2_ transition of Yb^3+^ was calculated by below formula,

6 $${\sigma }_{{ab}}^{{Yb}}\left({\rm{\lambda }}\right)=\frac{1}{2}\frac{\frac{1}{{\tau }_{{ex}}}-{\,A}_{4I13\to 4I15/2}^{{MD}}}{{A}_{4I13\to 4I15/2}^{{\prime} {ED}}}{\alpha }_{{Yb}}^{{\prime} }\left({\rm{\lambda }}\right)$$

where ${\alpha }_{{Yb}}^{{\prime} }\left({\rm{\lambda }}\right)$ is the spectral intensity for ^2^F_7/2_→^2^F_5/2_ transition of Yb^3+^ at wavelength ${\rm{\lambda }}$, ${{A}}_{4I13\to 4I15/2}^{{MD}}$ is magnetic dipole allowed transition rate for ^4^I_13/2_→^4^I_15/2_ transition and can be found in Table 1, ${A}_{4I13\to 4I15/2}^{{\prime} {ED}}$ is relative radiative electric dipole allowed transition rate for ^4^I_13/2_→^4^I_15/2_ transition and can be calculated directly from Eq. (S6). The reason for the insert of coefficient $\frac{1}{2}$ in Eq. (6) is that the Yb^3+^ concentration is two times the Er^3+^ concentration. By taking the corresponding data in Fig. S2 into Eq. (6), the emission cross-section ${\sigma }_{{ab}}^{{Yb}}\left({\rm{\lambda }}\right)$ was confirmed and plotted in Fig. S5 as solid curve. It should be mentioned that in Fig. S2 the absorption peak centered at 980 nm contains both contributions of the transition ^4^I_15/2_→^4^I_11/2_ of Er^3+^ and the transition ^2^F_7/2_→^2^F_5/2_ of Yb^3+^, and in Eq. (6) the ${\alpha }_{{Yb}}^{{\prime} }\left({\rm{\lambda }}\right)$ was derived by subtracting the spectral intensity of 5 mol% Er^3+^ single-doped sample from spectral intensity of 5 mol% Er^3+^/10 mol% Yb^3+^ co-doped sample. According to the above calculation procedure, the energy transfer rates for all the samples with different Yb^3+^ concentrations were calculated, and then the energy transfer efficiencies were calculated via following formula,

7 $${{\rm{\eta }}}_{{\rm{ET}}2}=\frac{{A}_{{et}}^{D\to A}}{{A}_{r}^{4I11/2}+{A}_{{nr}}^{4I11/2}+{A}_{{et}}^{D\to A}}$$

where ${A}_{r}^{4I11/2}$ is the sum of the radiative transition rates of Er^3+^ from ^4^I_11/2_ to ^4^I_13/2_ and ^4^I_15/2_ (the values are listed in Table 1), ${A}_{{nr}}^{4I11/2}$ is the nonradiative transition rate of ^4^I_11/2_ of Er^3+^ (the value is also listed in Table 1). The dependence of energy transfer efficiency on the Yb^3+^ is shown in Fig. 4c as solid squares, and the dashed curve shows the changing trend.

##### Quantum cutting efficiency

In the above sub-Sections, all the depopulation rates including radiative transition rates, nonradiative transition rates, and energy transfer rates are derived, therefore the quantum cutting efficiency ${{\rm{\eta }}}_{QC}$ of Er^3+^/Yb^3+^ co-doped NaY(WO_4_)_2_ phosphors can be calculated via following formula,

8 $${{\rm{\eta }}}_{QC}=\left\{{{\rm{\eta }}}_{{ET}1}+\left[{{\rm{\eta }}}_{{ET}1}+{{\rm{\eta }}}_{{Multi}}\bullet \left(1-{{\rm{\eta }}}_{{ET}1}\right)\right]\bullet {{\rm{\eta }}}_{{ET}2}\right\}\bullet {{\rm{\eta }}}_{{Yb}}$$

where ${{\rm{\eta }}}_{{Yb}}$ is the luminescence quantum efficiency of Yb^3+^ in NaY(WO_4_)_2_, ${{\rm{\eta }}}_{{Multi}}$ is the efficiency for populating ^4^I_11/2_ via cascade nonradiative relaxations of ^4^S_3/2_ (see NR1, NR2 and NR3 in Fig. 3e) and multi-step radiative transitions from ^2^H_112_, ^4^S_3/2_, ^4^F_9/2_, and ^4^I_9/2_ to ^4^I_11/2_. The ${{\rm{\eta }}}_{{Multi}}$ is dependent on the radiative and nonradiative transitions of Er^3+^ in NaY(WO_4_)_2_ but independent from Yb^3+^ concentration. The nonradiative relaxation rate of ^2^F_5/2_ of Yb^3+^ can be ignored owing to the large enough energy gap between ^2^F_7/2_ and ^2^F_5/2_. The energy transfer rate from Yb^3+^ to any luminescence centers or quenching centers can also affect the luminescence quantum efficiency of Yb^3+^. To evaluate the influence of energy transfer from Yb^3+^ to other centers, the fluorescence decays of Yb^3+^ were measured under 451 nm excitation and are shown in Fig. S6. It is found that the fluorescence decay does not change with Yb^3+^ concentration, therefore ${{\rm{\eta }}}_{{Yb}}$ is taken as 1 in calculating the quantum cutting efficiency. In addition, ${{\rm{\eta }}}_{{Multi}}$ value can be calculated by using the radiative transition rates and nonradiative relaxation rates in Table 1, and the value of ${{\rm{\eta }}}_{{Multi}}$ is found to be 22.5%. In the calculation it is found that the contribution of the multi-step radiative transitions is very small since the radiative transition branching ratios from ^2^H_11/2_, ^4^S_3/2_, ^4^F_9/2_ and ^4^I_9/2_ to ^4^I_11/2_ are very small. Based on the above analyses, the quantum cutting efficiencies for all the samples with different Yb^3+^ concentrations were calculated, and the dependence of the quantum cutting efficiency on Yb^3+^ concentration is shown in Fig. 4c as solid circles. From Fig. 4c it can be seen that around 173% of quantum cutting efficiency can be realized in Er^3+^/Yb^3+^ co-doped NaY(WO_4_)_2_ phosphor when the Er^3+^ and Yb^3+^ concentrations are 5 mol% and 50 mol%, thus indicating Er^3+^/Yb^3+^ co-doped NaY(WO_4_)_2_ phosphor is an excellent quantum cutting material. It should be mentioned that the quantum cutting efficiency calculated in this work is different from the experimentally measured efficiency. The calculated efficiency is internal quantum efficiency that means it does not conclude the transmission efficiency of excitation light and emission light in the host material. Therefore, the calculated quantum cutting efficiency is usually larger than or close to the experimentally measured efficiency.

### 980 nm up-conversion emission under 1550 nm excitation

It is well known that the Er^3+^ doped materials can convert the 1550 nm photons into solar-cell-absorbable photons including visible and near-infrared photons via the so-called frequency upconversion processes. In the previous Section it was discovered that the energy transfer ET2 from Er^3+^ to Yb^3+^ is very efficient in the co-doped samples. Therefore, it is expected that the energy transfer ET2 could also take effect and result in efficient Yb^3+^ emission in the upconversion process when the co-doped samples are excited at 1550 nm.

#### Concentration effects of upconversion luminescence

**Fig. 5:**
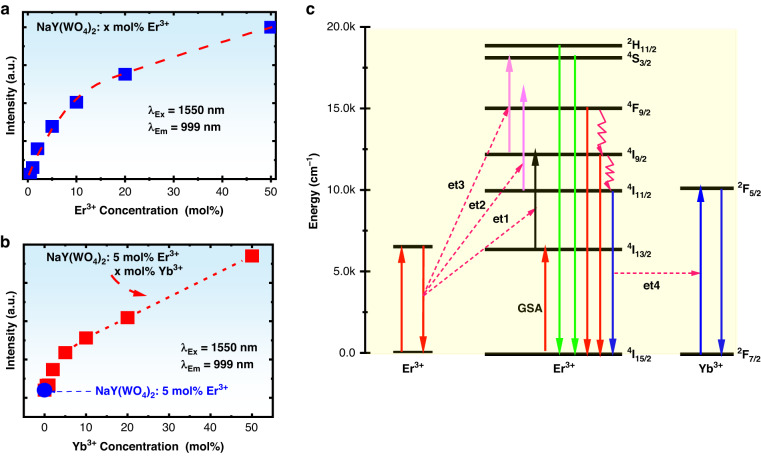
Concentration effects of upconversion luminescence and upconversion mechanism. **a** Dependence of integrated upconversion intensity on Er^3+^ concentration; **b** Dependence of integrated upconversion intensity on Yb^3+^ concentration. **c** Up-conversion mechanisms for Er^3+^ single-doped and Er^3+^/Yb^3+^ co-doped NaY(WO_4_)_2_ phosphors

In this section the concentration dependence of upconversion emission intensity will be examined. Fig. S7 shows the upconversion emission spectra in the wavelength range from 400 nm to 1100 nm for the samples with different Er^3+^ concentration, and the insert shows the enlarged upconversion emission spectra in the wavelength range from 400 nm to 900 nm. It can be seen that under 1550 nm excitation the Er^3+^ single-doped NaY(WO_4_)_2_ phosphors mainly emit 999 nm infrared light corresponding ^4^I_11/2_→^4^I_15/2_ transition of Er^3+^, and the other upconversion emissions corresponding ^2^H_11/2_/^4^S_3/2_→^4^I_15/2_, ^4^F_9/2_→^4^I_15/2_ and ^4^I_9/2_→^4^I_15/2_ are extremely weak. The dispersed solid squares in Fig. 5a show the dependence of 999 nm upconversion intensity on the Er^3+^ doping concentration. It is found that the upconversion emission intensity monotonically increases but its growing rate decreases with increasing Er^3+^ concentration. As a compromise between the quantum cutting and the upconversion, the concentration selection of 5 mol% Er^3+^ is also reasonable for further introducing Yb^3+^. Figure S8 shows the upconversion emission spectra for the samples doped with fixed 5 mol% Er^3+^ and varied Yb^3+^ concentrations (0, 0.5, 1, 2, 5, 10, 20, and 50 mol%). It can be seen that nearly pure infrared emission of Yb^3+^ at 999 nm accompanying with very weak visible emissions was observed and the infrared emission intensity increases with increasing Yb^3+^ concentration. The insert of Fig. S8 shows the enlarged spectra ranging from 400 nm to 900 nm. Figure 5b shows the dependence of the infrared emission integrated intensity on the Yb^3+^ concentration. From Fig. 5b it can be seen that the Yb^3+^ introduction greatly increases the infrared emission, and the infrared upconversion intensity of 5 mol% Er^3+^/50 mol% Yb^3+^ co-doped phosphor is 20 times higher than the intensity of 5 mol% Er^3+^ single-doped phosphor. The concentration quenching for the upconversion process is different from the down-shifting process since the concentration quenching depends on the excitation wavelength. Different excitation wavelengths result in different populating and depopulating pathways.

#### Excitation power dependences of upconversion luminescence

From Figs. S7 and S8 it can be found that the upconversion spectra are almost not changed except for the spectral intensities, thus indicating the upconversion mechanisms are not changed with doping concentration. To discover the upconversion mechanism, the upconversion emission spectra for 5 mol% Er^3+^ single-doped NaY(WO_4_)_2_ phosphor excited at varied excitation power were measured. In this work, the excitation power of the 1550 nm laser was controlled by changing working current of the laser. Fig. S9a shows the upconversion emission spectra measured at different working currents. The insert of Fig. S9a displays the enlarged upconversion spectra ranging from 500 nm to 600 nm for ^2^H_11/2_/^4^S_3/2_→^4^I_15/2_ transitions. The relation between the fluorescence intensity ratio of ^2^H_11/2_→^4^I_15/2_ to ^4^S_3/2_→^4^I_15/2_ and the working current is depicted in Fig. S9b. It can be seen that the fluorescence intensity ratio does not change with increasing working current, thus implying the sample temperature was kept as a constant. This constant temperature means that there is no fluorescence temperature quenching caused by laser irradiation in the spectral measurements. Therefore, the excitation-power-dependent spectra are reliable for deducing the upconversion mechanism. The dependence of the integrated upconversion intensity for ^4^I_11/2_→^4^I_15/2_ emission on the working current was derived from the spectra in Fig. S9a and is shown in Fig. S9c. The data in Fig. S9 was fit to the following function^52^.

9 $$I\left(i\right)=a{\left(i-{i}_{0}\right)}^{n}$$

where $I\left(i\right)$ is the integrated upconversion intensity at working current $i$, *a* is a constant, $n$ is the excitation photon number needed for emitting one 999 nm photon, ${i}_{0}$ is the threshold current of the 1550 nm laser. From the fitting process, the n was confirmed to be 2.13 which is close the theoretical value for the two-photon process, thus implying the 999 nm upconversion emission is a two-photon process.

In an analogical way, the upconversion spectra for the samples doped with 5 mol% Er^3+^ and 10 mol% Yb^3+^ were also measured under 1550 nm excitation and are shown in Fig. S10a. The unchanged fluorescence ratios at different currents were also observed as seen in Fig. S10b. The similar fitting operation was also carried out for the data in Fig. S10c, and a two-photon process was also confirmed to be dominant for Yb^3+^ upconversion emission.

#### Upconversion mechanism

From the upconversion spectra and the excitation-power-dependent upconversion intensity, the possible upconversion mechanisms for Er^3+^ and Er^3+^/Yb^3+^ doped NaY(WO_4_)_2_ phosphors are portrayed in Fig. 5c. In Er^3+^ single-doped NaY(WO_4_)_2_ phosphors under 1550 nm excitation the ^4^I_13/2_ can be populated via ground state absorption. The ^4^I_9/2_ is mainly populated via an energy transfer et1 since the excited state absorption ^4^I_13/2_→^4^I_9/2_ is rather weak owing to the large energy mismatch and small transition rate of ^4^I_13/2_→^4^I_9/2_ (see Table 1). ^4^I_9/2_ is mainly depopulated via a nonradiative relaxation to ^4^I_11/2_, therefore the intense ^4^I_11/2_→^4^I_15/2_ upconversion emission could be observed in the Er^3+^ single-doped samples. The ^4^F_9/2_ is mainly populated via an energy transfer et2, but the energy transfer rate is relatively weak owing to the large energy mismatch though the ^4^I_11/2_ is heavily populated via a nonradiative relaxation from ^4^I_9/2_ to ^4^I_11/2_. Therefore, the red upconversion emission is weak. The ^4^S_3/2_ is populated via an energy transfer et3, but the population of ^4^I_9/2_ is less owing to the strong nonradiative transition. Therefore, the green emissions of ^4^S_3/2_/^2^H_11/2_→^4^I_15/2_ are also weak.

When Yb^3+^ is introduced, the population pathways of Er^3+^ under 1550 nm excitation will be the same as in Er^3+^ single-doped NaY(WO_4_)_2_ phosphors. It should be mentioned that we have not observed any emissions in the Yb^3+^ single-doped NaY(WO_4_)_2_ phosphors (even with high concentration). However, Yb^3+^ will accept the energy transferred from Er^3+^ since the energy transfer efficiency for et4 in Fig. 5c is very large as discovered in the above Section where it was stated as ET2. Therefore, the ^4^I_11/2_→^4^I_15/2_ emission decreases but ^2^F_5/2_→^2^F_7/2_ of Yb^3+^ increases. These upconversion mechanisms tell us that both Er^3+^ and Er^3+^/Yb^3+^ doped NaY(WO_4_)_2_ phosphors exhibit strong near infrared emissions from ^4^I_11/2_→^4^I_15/2_ of Er^3+^ and ^2^F_5/2_→^2^F_7/2_ of Yb^3+^ that indicates the studied phosphors are good light conversion candidate for silicon-based solar cell applications. In this work, the quantum cutting and upconversion processes were individually studied, but in practical application these two processes co-happen at the same time. The quantum cutting and upconversion processes are dependent on the energy level distribution of the luminescence centers. The quantum cutting and upconversion processes can exist as long as the energy level structure does not change. However, the efficiencies of the quantum cutting and upconversion processes are different in these two cases: co-happen and solo-exist because the population processes in the co-happened case are different from the studied cases.

### Optical temperature sensing

When the NaY(WO_4_)_2_:Er^3+^/Yb^3+^ is introduced into the silicon-based photovoltaic system, the spectral conversions including quantum cutting and upconversion could improve the photoelectric conversion efficiency, and simultaneously the system temperature could also be read out via a fluorescence intensity ratio technique as we stated in the Introduction section. Therefore, in this section, the temperature sensing performance of NaY(WO_4_)_2_:Er^3+^/Yb^3+^ will be assessed.

#### Temperature sensing properties based on fluorescence intensity ratio

**Fig. 6:**
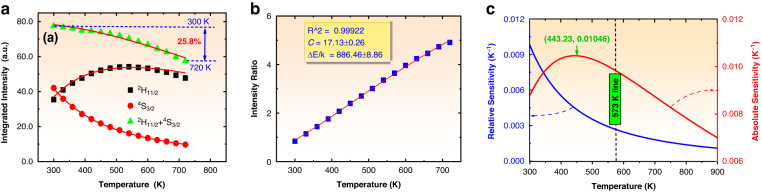
Luminescence temperature quenching and optical temperature sensing. Fluorescence thermal properties of 5 mol% Er^3+^ and 10 mol% Yb^3+^ co-doped NaY(WO_4_)_2_ phosphor **a** temperature-dependent up-conversion emission intensities: ■ for ^2^H_11/2_→^4^I_15/2_, ● for ^4^S_3/2_→^4^I_15/2_, and ▲ for the sum of both; **b** fluorescence intensity ratio of ^2^H_11/2_→^4^I_15/2_, to ^4^S_3/2_→^4^I_15/2_: Dispersed solid squares are the experimental data, solid curve is the fitting curve; **c** Functions of relative and absolute sensitivities versus temperature

For a certain Er^3+^ doped system, the temperature sensing properties are usually independent from doping concentration unless the optical transition property of Er^3+^ changes greatly with doping concentration^53^. To examine the temperature sensing, the 5 mol% Er^3+^ and 10 mol% Yb^3+^ co-doped NaY(WO_4_)_2_ phosphor was selected for study and its green emission spectra were measured at different temperatures from 300 K to 720 K under 378 nm excitation and are shown in Fig. S11. The integrated emission intensities of ^2^H_11/2_→^4^I_15/2_ and ^4^S_3/2_→^4^I_15/2_ transitions were calculated. The solid squares, solid circles, and triangles in Fig. 6a show the temperature-dependent fluorescence intensities for ^2^H_11/2_→^4^I_15/2_ and ^4^S_3/2_→^4^I_15/2_ transitions and their sum. It can be seen that ^4^S_3/2_→^4^I_15/2_ emission intensity monotonically decreases with increasing sample temperature, and this decrease can be attributed to its two enhanced depopulation rates, namely nonradiative relaxation rate to ^4^F_9/2_ and thermal population rate to ^2^H_11/2_. The ^2^H_11/2_→^4^I_15/2_ emission intensity increases first and then decreases with increasing temperature that is caused by two competing population and depopulation processes. The population process is the thermal excitation of ^4^S_3/2_ which leads to enhancing emission intensity, and the depopulation process is the decrease of the sum population of ^2^H_11/2_ and ^4^S_3/2_ due to nonradiative relaxation of ^4^S_3/2_ which leads to depressing the emission intensity. From Fig. 6a it can be found that the studied phosphors exhibit excellent temperature quenching that the sum of two green emission intensities shows a decrease of only 25.8% when the temperature increases from 300 K to 720 K. This excellent temperature quenching performance of the studied phosphors benefits the application in solar cells since the solar cells are often work at high temperatures.

The solid squares in Fig. 6b show the fluorescence intensity ratio of ^2^H_11/2_→^4^I_15/2_ transition to ^4^S_3/2_→^4^I_15/2_ transition. The data in Fig. 6b was fit to the following formula^54,55^

10 $${FIR}=\frac{{I}_{H}}{{I}_{S}}=C{e}^{-\frac{\triangle E}{{kT}}}$$

where ${I}_{H}$ and ${I}_{S}$ are the green emission intensities of ^2^H_11/2_→^4^I_15/2_ and ^4^S_3/2_→^4^I_15/2_ transitions, $\triangle E$ is the energy distance between ^2^H_11/2_ and ^4^S_3/2_. The parameter values of $C$ and $\triangle E/k$ were determined to be 17.13 and 886.46 K via fitting Eq. (10) to the data in Fig. 6b. Once these parameters are confirmed, the NaY(WO_4_)_2_:Er^3+^/Yb^3+^ can be used as a temperature probe. To evaluate the temperature sensing performance the absolute (${S}_{A}$) and relative (${S}_{R}$) sensitivities are calculated via the following functions^54,55^

11 $${S}_{A}=\left|\frac{d\left({FIR}\right)}{{dT}}\right|=C\frac{\triangle E}{k{T}^{2}}\exp \left(-\frac{\triangle E}{{kT}}\right)$$

12 $${S}_{R}=\frac{{S}_{A}}{{FIR}}=\left|\frac{1}{{FIR}}\frac{d\left({FIR}\right)}{{dT}}\right|=\frac{\triangle E}{k{T}^{2}}$$

By taking the parameter values of $C$ and $\triangle E/k$ into Eqs. (11) and (12), the absolute and relative sensitivities are derived and are plotted in Fig. 6c. The maximum value of ${S}_{A}^{\max }$ and its corresponding temperature value ${T}_{\max }$ can be determined via a simple mathematical treatment to be ${S}_{A}^{\max }=4\frac{{Ck}}{{e}^{2}\triangle E}$ and ${T}_{\max }=\frac{\triangle E}{2k}$^54^ which are marked in Fig. 6c. Usually, the solar cells operate at the temperature lower than 573 K (300 °C) since at the temperature higher than 573 K the solar cells will be damaged. From Fig. 6c it can be seen that in the temperature range from room temperature 300 K (27 °C) to 573 K (300 °C) the studied NaY(WO_4_)_2_:Er^3+^/Yb^3+^ works in the status with the best absolute and relative sensitivities in its possible working temperature range.

#### Temperature quenching mechanism of green emissions of Er^3+^ in NaY(WO_4_)_2_ phosphors

In Fig. 6a the temperature quenching of green emissions of Er^3+^ in NaY(WO_4_)_2_ was observed, but the temperature quenching mechanism is not clear so far. If the nonradiative relaxation of ^4^S_3/2_ is the main depopulation route, then the fluorescence intensity ${I}_{s}$ of ^4^S_3/2_ can be theoretically expressed as follows,

13 $${I}_{S}=a\frac{{A}_{r}}{{A}_{{all}}+{W}_{{nr}}(0){\left[1-\exp \left(-{hv}/{kT}\right)\right]}^{-p}}$$

where ${A}_{r}$ is radiative transition rate of ^4^S_3/2_→^4^I_15/2_ transition, $a$ is temperature-independent constant, ${A}_{{all}}$ is the sum of all depopulation rates of ^4^S_3/2_ except for the nonradiative relaxation rate, and ${W}_{{nr}}(0){\left[1-\exp \left(-{hv}/{kT}\right)\right]}^{-p}$ is the nonradiative relaxation rate of ^4^S_3/2_. Here, ${W}_{{nr}}(0)$ is the nonradiative relaxation rate at a temperature infinitely close to 0 K. $v$ is the effective phonon frequency, $p$ stands for the generated phonon number in the nonradiative relaxation process. By combining Eqs. (10) and (13), it can be derived that

14 $${I}_{S}=b\frac{{A}_{r}}{{A}_{{all}}+{W}_{{nr}}(0){\left[1-\exp \left(-{hv}/{kT}\right)\right]}^{-p}}{\rm{\cdot }}\frac{1}{1+C{e}^{-\frac{\triangle E}{{kT}}}}$$

15 $${I}_{H}=b\frac{{A}_{r}}{{A}_{{all}}+{W}_{{nr}}(0){\left[1-\exp \left(-{hv}/{kT}\right)\right]}^{-p}}{\rm{\cdot }}\frac{C{e}^{-\frac{\triangle E}{{kT}}}}{1+C{e}^{-\frac{\triangle E}{{kT}}}}$$

where $b$ is a constant independent from temperature. Eqs. (14) and (15) were fit to the data for ^2^H_11/2_→^4^I_15/2_ and ^4^S_3/2_→^4^I_15/2_ transitions in Fig. 6a. The solid curves in Fig. 6a are the fitting curves, and it can be seen that Eqs. Eqs. (14) and (15) fit in well with the experimental data. Moreover, the function of ${I}_{H}+{I}_{S}$ was fit to the data for fluorescence sum of ^2^H_11/2_→^4^I_15/2_ and ^4^S_3/2_→^4^I_15/2_ transitions, and the solid curve shows the fitting curve. In this fitting process the parameters $C$ and $\triangle E/k$ were fixed as derived from Eq. (10), and the parameters ${hv}/k$ and $p$ were taken as free parameters. From two fitting processes for ^2^H_11/2_→^4^I_15/2_ and ^4^S_3/2_→^4^I_15/2_ transitions, the parameters ${hv}/k$ and $p$ were determined to be 1245.0 K and 3.6. Therefore, the maximum phonon energy ${{dE}}_{\omega }$ of the NaY(WO_4_)_2_ can be estimated to be 889.3 cm^-1^ which is very close the experimental value of 900 cm^-1^. From Table 1 it is known that the energy difference ${dE}$ between ^4^S_3/2_ and ^4^F_9/2_ is around 3091.83 cm^-1^, therefore the generated phonon number in the multiphonon nonradiative relaxation of ^4^S_3/2_ is ${dE}/{{dE}}_{\omega }\approx 3.4$ which is close to the $p$ value of 3.6, thus implying fluorescence quenching of ^2^H_11/2_/^4^S_3/2_→^4^I_15/2_ is caused by the nonradiative relaxation of ^4^S_3/2_.

## Conclusions

In summary, tri-functionalization including quantum cutting, infrared upconversion, and temperature detection was realized in Er^3+^/Yb^3+^ co-doped NaY (WO_4_)_2_ phosphors. Based on the optical spectroscopic analyses, the quantum cutting mechanism was discovered, and the photon splitting process includes two-step energy transfer processes, namely, ^4^S_3/2_ + ^2^F_7/2_ →^4^I_11/2_ + ^2^F_5/2_ and ^4^I_11/2_ + ^2^F_7/2_→ ^4^I_15/2_ + ^2^F_5/2_. Furthermore, the radiative transition rates for all interested transitions of Er^3+^ were calculated in the framework of Judd-Ofelt theory, and the nonradiative transition rates for all concerned energy levels were also derived according to the energy gap law. Starting from the radiative transition rates, nonradiative transition rates, and experimental fluorescence lifetimes of correlated energy levels, the quantum cutting efficiencies were calculated, and the concentration-dependent maximum quantum cutting rate was found to be as high as 173%. Under 1550 nm excitation, the near infrared emissions of ^4^I_11/2_→^4^I_15/2_ and ^2^F_5/2_→^2^F_7/2_ from Er^3+^ single-doped and Er^3+^/Yb^3+^ co-doped NaY(WO_4_)_2_ phosphors were observed, respectively. It was found that all samples with various doping concentrations showed nearly pure-color near infrared emissions, and the possible upconversion mechanisms were proposed. The studied materials displayed excellent thermal quenching of ^4^S_3/2_→^4^I_15/2_ emission that is beneficial to the quantum cutting. The thermal quenching mechanism was assigned to the nonradiative transition of ^4^S_3/2_. Moreover, the temperature sensing study proved that the Er^3+^/Yb^3+^ co-doped NaY(WO_4_)_2_ phosphors presented excellent temperature sensing performance. All these results imply that Er^3+^/Yb^3+^ co-doped NaY(WO_4_)_2_ phosphors are excellent light-converted and temperature sensing material having potential application in silicon-based solar cells to improve photovoltaic performance.

### Supplementary information

Supplemental Material

## References

[1] Höök M, Tang X (2013). Depletion of fossil fuels and anthropogenic climate change-a review. Energy Policy.

[2] Tamilselvan P, Nallusamy N, Rajkumar S (2017). A comprehensive review on performance, combustion and emission characteristics of biodiesel fuelled diesel engines. Renew. Sustain. Energy Rev..

[3] Lee J (2023). Hybrid renewable energy systems involving thermochemical conversion process for waste-to-energy strategy. Chem. Eng. J..

[4] Bie PJ (2023). A review and evaluation of nonroad diesel mobile machinery emission control in China. J. Environ. Sci..

[5] Raheem I (2023). Rapid growth of MXene-based membranes for sustainable environmental pollution remediation. Chemosphere.

[6] González-Torres M (2022). A review on buildings energy information: trends, end-uses, fuels and drivers. Energy Rep..

[7] Abdelfattah A (2023). Microalgae-based wastewater treatment: mechanisms, challenges, recent advances, and future prospects. Environ. Sci. Ecotechnol..

[8] Lin YH (2023). Alleviating the self-discharge and enhancing the polysulphides conversion kinetics with LaCO_3_OH nanocrystals decorated hierarchical porous carbon. Chem. Eng. J..

[9] Kumar A (2023). A review on S-scheme and dual S-scheme heterojunctions for photocatalytic hydrogen evolution, water detoxification and CO_2_ reduction. Fuel.

[10] Salamah T (2022). Effect of dust and methods of cleaning on the performance of solar PV module for different climate regions: comprehensive review. Sci. Total Environ..

[11] Wu N (2023). Efficient furan-bridged dibenzofulvene-triphenylamine hole transporting materials for perovskite solar cells. Mater. Adv..

[12] Zhao JL (2023). Photochromic crystalline hybrid materials with switchable properties: recent advances and potential applications. Coord. Chem. Rev..

[13] Ma ZX (2023). Efficient decontamination of organic pollutants from wastewater by covalent organic framework-based materials. Sci. Total Environ..

[14] Liao GF (2022). Z-scheme systems: from fundamental principles to characterization, synthesis, and photocatalytic fuel-conversion applications. Phys. Rep..

[15] Mehta N (2023). Down-conversion of a single photon as a probe of many-body localization. Nature.

[16] Arduini F (2020). Carbon black as an outstanding and affordable nanomaterial for electrochemical (bio)sensor design. Biosens. Bioelectron..

[17] Tian N (2022). Layered bismuth-based photocatalysts. Coord. Chem. Rev..

[18] Petrov V (2015). Frequency down-conversion of solid-state laser sources to the mid-infrared spectral range using non-oxide nonlinear crystals. Prog. Quant. Electron..

[19] Hola K (2014). Carbon dots-Emerging light emitters for bioimaging, cancer therapy and optoelectronics. Nano Today.

[20] Pulli E, Rozzi E, Bella F (2020). Transparent photovoltaic technologies: current trends towards upscaling. Energy Convers. Manag..

[21] Jia YT, Alva G, Fang GY (2019). Development and applications of photovoltaic–thermal systems: a review. Renew. Sustain. Energy Rev..

[22] Alami AH (2022). Management of potential challenges of PV technology proliferation. Sustain. Energy Technol. Assess..

[23] Mojiri A (2013). Spectral beam splitting for efficient conversion of solar energy-a review. Renew. Sustain. Energy Rev..

[24] Raijmakers LHJ (2019). A review on various temperature-indication methods for Li-ion batteries. Appl. Energy.

[25] Abram C, Fond B, Beyrau F (2018). Temperature measurement techniques for gas and liquid flows using thermographic phosphor tracer particles. Prog. Energy Combust. Sci..

[26] Taylor NAS, Tipton MJ, Kenny GP (2014). Considerations for the measurement of core, skin and mean body temperatures. J. Therm. Biol..

[27] Roy A (2023). The impact of pure and mixed self-activated YXO_4_ phosphor materials (X=V, Nb and Ta) on downshifting and quantum cutting emission behaviours of Ln^3+^ doped (Ln^3+^=Ho^3+^ and Yb^3+^) ions. Ceram. Int..

[28] Mishra NK (2023). Probing multimodal light emission from Tb^3+^/Yb^3+^-doped garnet nanophosphors for lighting applications. Phys. Chem. Chem. Phys..

[29] Li DC (2023). Quantum cutting in KGd(CO_3_)_2_: Tb^3+^ green phosphor. Nanomaterials.

[30] Balaji S (2017). Insights into Er^3+^↔Yb^3+^ energy transfer dynamics upon infrared ~1550 nm excitation in a low phonon fluoro-tellurite glass system. J. Lumin..

[31] Wegh RT (1999). Visible quantum cutting in LiGdF_4_: Eu^3+^ through downconversion. Science.

[32] Ye S (2008). Enhanced cooperative quantum cutting in Tm^3+^-Yb^3+^ codoped glass ceramics containing LaF_3_ nanocrystals. Opt. Express.

[33] Chen DQ (2008). Quantum cutting downconversion by cooperative energy transfer from Ce^3+^ to Yb^3+^ in borate glasses. J. Appl. Phys..

[34] Roh JYD (2023). Negative thermal quenching in quantum-cutting Yb^3+^-doped CsPb(Cl_1-*x*_Br_*x*_)_3_ Perovskite nanocrystals. ACS Nano.

[35] Zi L (2023). X-ray quantum cutting scintillator based on CsPbCl_*x*_Br_3-*x*_: Yb^3+^ single crystals. Laser Photon. Rev..

[36] Zhao Y (2020). Optical temperature sensing of up-conversion luminescent materials: fundamentals and progress. J. Alloys Compd.

[37] Zhu L, Zeng W (2017). Room-temperature gas sensing of ZnO-based gas sensor: a review. Sens. Actuat. A: Phys..

[38] Chen ZL, Galli M, Gallavotti A (2022). Mechanisms of temperature-regulated growth and thermotolerance in crop species. Curr. Opin. Plant Biol..

[39] de Mendívil JM (2015). Judd-Ofelt analysis and transition probabilities of Er^3+^ doped KY_1-*x*-*y*_Gd_*x*_Lu_*y*_(WO_4_)_2_ crystals. J. Lumin..

[40] Zhang LZ (2018). Crystal growth, spectroscopy and first laser operation of a novel disordered tetragonal Tm: Na_2_La_4_(WO_4_)_7_ tungstate crystal. J. Lumin..

[41] Zhang LZ (2017). Crystal growth, optical spectroscopy and laser action of Tm^3+^-doped monoclinic magnesium tungstate. Opt. Express.

[42] Auzel F (2002). A fundamental self-generated quenching center for lanthanide-doped high-purity solids. J. Lumin..

[43] Liang YJ (2016). New function of the Yb^3+^ ion as an efficient emitter of persistent luminescence in the short-wave infrared. Light Sci. Appl..

[44] Zhang Y (2022). Blue LED-pumped intense short-wave infrared luminescence based on Cr^3+^-Yb^3+^-co-doped phosphors. Light Sci. Appl..

[45] Green, M. A. *Solar Cells: Operating Principles, Technology, and System Applications* (Kensington: University of New South Wales, 1998).

[46] Zhang YQ (2018). A universal approach for calculating the Judd–Ofelt parameters of RE^3+^ in powdered phosphors and its application for the β-NaYF_4_: Er^3+^/Yb^3+^ phosphor derived from auto-combustion-assisted fluoridation. Phys. Chem. Chem. Phys..

[47] Luo MY (2022). Optical transition properties, internal quantum efficiencies, and temperature sensing of Er^3+^ doped BaGd_2_O_4_ phosphor with low maximum phonon energy. J. Am. Ceram. Soc..

[48] Sha XZ (2021). Pre-assessments of optical transition, gain performance and temperature sensing of Er^3+^ in NaLn(MoO_4_)_2_ (Ln = Y, La, Gd and Lu) single crystals by using their powder-formed samples derived from traditional solid state reaction. Opt. Laser Technol..

[49] de Sousa DF (2000). Energy transfer and the 2.8 - *μ*m emission of Er^3+^-and Yb^3+^- doped low silica content calcium aluminate glasses. Phys. Rev. B.

[50] Pecoraro E (1999). Evaluation of the energy transfer rate for the Yb^3+^: Pr^3+^ system in lead fluoroindogallate glasses. J. Appl. Phys..

[51] Kim M (2019). Methylammonium chloride induces intermediate phase stabilization for efficient perovskite solar cells. Joule.

[52] Zhou TM (2015). Concentration effect and temperature quenching of upconversion luminescence in BaGd_2_ZnO_5_: Er^3+^/Yb^3+^ phosphor. J. Rare Earths.

[53] Liu LT (2018). Dependence of optical temperature sensing and photo-thermal conversion on particle size and excitation wavelength in *β*-NaYF_4_: Yb^3+^, Er^3+^ nanoparticles. J. Alloys Compd.

[54] Li YC (2019). A temperature self-monitoring NaYF_4_: Dy^3+^/Yb^3+^@NaYF_4_: Er^3+^/Yb^3+^ core-shell photothermal converter for photothermal therapy application. Results Phys..

[55] Collins SF (1998). Comparison of fluorescence-based temperature sensor schemes: theoretical analysis and experimental validation. J. Appl. Phys..

