# Supplementary material for: Near infrared emissions from both high efficient quantum cutting (173%) and nearly-pure-color upconversion in NaY(WO4)2:Er3+/Yb3+ with thermal management capability for silicon-based solar cells
Source: Light Sci Appl. 2024 Jan 16;13:17. doi: 10.1038/s41377-023-01365-2 (PMC10789824; doi:10.1038/s41377-023-01365-2)
Supplement: Supplementary file 1 — Supplemental Material [file 41377_2023_1365_MOESM1_ESM.docx]

**Supplementary Information for ‘Near Infrared Emissions from Both High Efficient Quantum Cutting (173%) and Nearly-Pure-Color Upconversion in NaY(WO_4_)_2_:Er^3+^/Yb^3+^ with Thermal Management Capability for Silicon-Based Solar Cells’**

Duan Gao, Baojiu Chen*, Xuezhu Sha, Yuhang Zhang, Xin Chen, Li Wang, Xizhen Zhang, Jinsu Zhang, Yongze Cao, Yichao Wang, Lei Li, Xiangping Li, Sai Xu, Hongquan Yu, Lihong Cheng

**Supplementary Information**

**Paper title:** Near Infrared Emissions from Both High Efficient Quantum Cutting (173%) and Nearly-Pure-Color Upconversion in NaY(WO_4_)_2_:Er^3+^/Yb^3+^ with Thermal Management Capability for Silicon-Based Solar Cells

**Authors:** Duan Gao, Baojiu Chen*, Xuezhu Sha, Yuhang Zhang, Xin Chen, Li Wang, Xizhen Zhang, Jinsu Zhang, Yongze Cao, Yichao Wang, Lei Li, Xiangping Li, Sai Xu, Hongquan Yu, Lihong Cheng

**Affiliation:** School of Science, Dalian Maritime University, Dalian 116026, Liaoning, PR China

**<Fig. S1>**


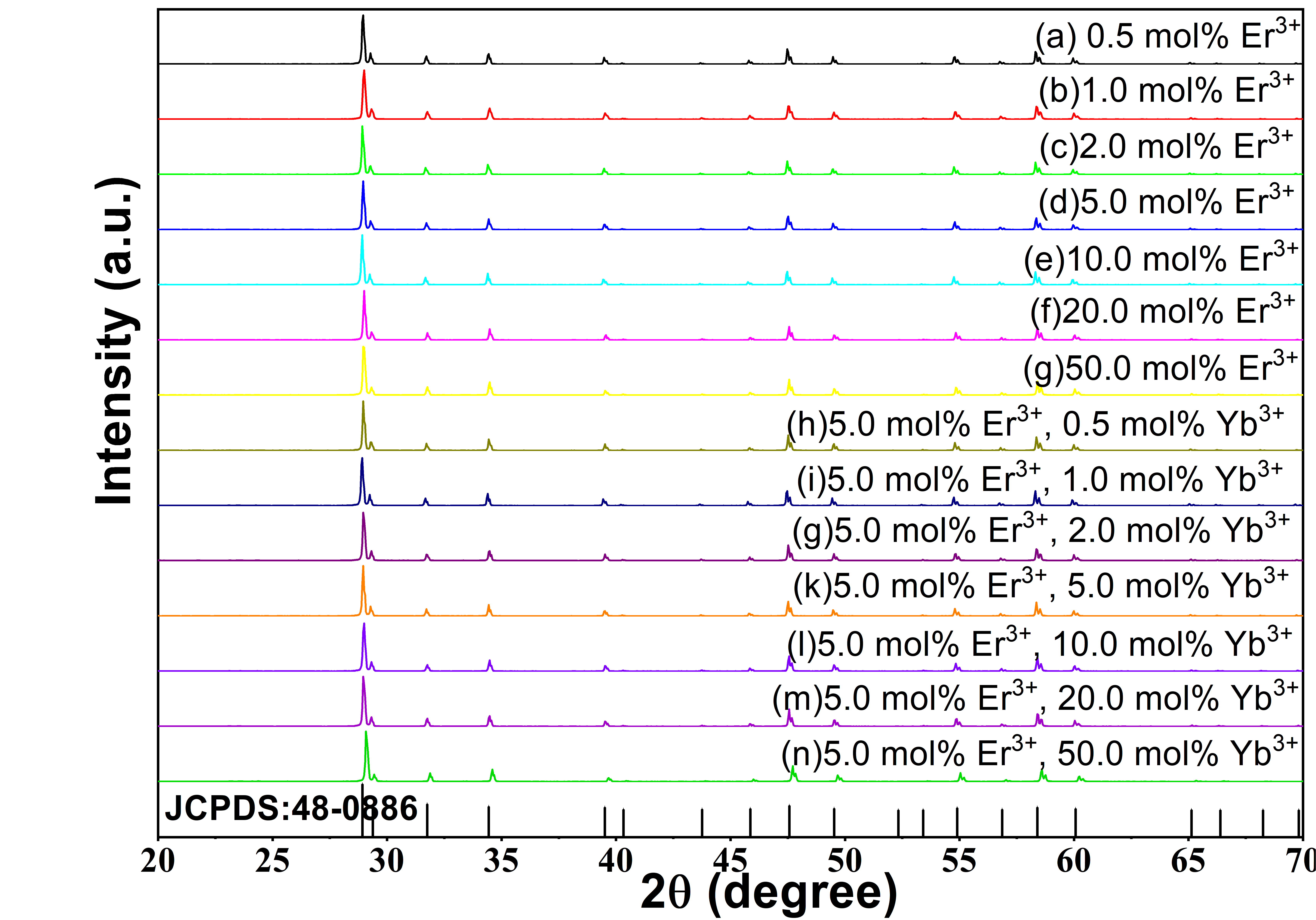


Fig. S1 XRD patterns for all obtained NaY(WO_4_)_2_ phosphors and the pattern reported in JCPDS card No. 48-0886 (on the bottom).

**<Judd-Ofelt calculation>**

First, the diffuse reflection spectrum (Fig. S2 (a)) of 5 mol% Er^3+^ and 10 mol% Yb^3+^ co-doped NaY(WO_4_)_2_ phosphor was transformed into relative absorption spectrum (Fig. S2 (b)) with arbitrary intensity via below Kubelka-Munk function ^40,41^,

$\alpha^{'}\left( v \right)=C\frac{\left[ 1-R\left( v \right) \right]^{2}}{2R\left( v \right)}$ (S1)

In Eq. (S1), $\alpha^{'}\left( v \right)$ and $R(v)$ are the relative absorption intensity and the reflection ratio at wavenumber *v*, and $C$ is a constant independent from wavenumber. The relative experimental oscillator strength $f_{exp}^{'}$ was derived by taking the data for a certain absorption peak in the relative absorption spectrum into Eq. (S2) below.

$f_{exp}^{'}=\frac{mc^{2}}{\pi e^{2}}\int\alpha^{'}\left( v \right)dv$ (S2)

In Eq. (S2), $m$ is the electron mass, $c$ is the speed of light in vacuum, $\pi$ is circumference ratio and $e$ is the electron charge. For every observed transition in Fig. S1, let the relative experimental oscillator strength equal the theoretical oscillator strength $f_{th}^{ED}$ expressed as following Eq. (S3), then a set of equations is established when taking all transitions into account.

$f_{th}^{ED}=\frac{8\pi^{2}mcv}{3h\left( 2J+1 \right)}\frac{\left( n^{2}+2 \right)^{2}}{9n}\sum_{\lambda=2,4,6} {{\Omega'}_{\lambda}\left| \left\langle\left( S,L \right)J\left| U^{\lambda} \right|\left( S',L' \right)J' \right\rangle\right|}^{2}$ (S3)

Here, $n$ is index of refraction, $h$ is the Planck constant, $J$ is equal to 15/2 for Er^3+^, ${\Omega'}_{\lambda}$(λ=2,4,6) are the relative Judd-Ofelt parameters, $\left\langle\left( S,L \right)J\left| U^{\lambda} \right|\left( S',L' \right)J' \right\rangle$ is the reduced matrix element for the transition from $\left\langle\left( S,L \right)J \right.|$ to $|\left. \left( S',L' \right)J' \right\rangle$. In this work, total six transitions from ^4^I_15/2_ to ^4^I_9/2_, ^4^F_9/2_, ^4^S_3/2_, ^2^H_11/2_, ^4^F_7/2_, and ^4^G_11/2_ are involved in the calculation. The relative experimental and theoretical oscillator strengths are listed in Table S1 together with the standard deviation. From the standard deviation and the comparison between the relative experimental and theoretical oscillator strengths, it confirms that the calculation results are reliable. The relative Judd-Ofelt parameters are ${\Omega'}_{2}=$ 1.44×10^-21^, ${\Omega'}_{4}=$ 1.70×10^-22^, and ${\Omega'}_{6}=$ 8.23×10^-24^. In order to obtain the real Judd-Ofelt parameters, the fluorescence decay for ^4^I_13/2_→^4^I_15/2_ transition of 0.5 mol% Er^3+^ doped NaY(WO_4_)_2_ phosphor was measured and is shown in Fig. S3 in the supporting information file. The fluorescence lifetime $\tau_{ex}$was derived to be 4.03 ms which is taken as the reciprocal of the radiative transition rate of ^4^I_13/2_→^4^I_15/2_ transition since the nonradiative transition rate and energy transfer rate can be ignored in the present case. For ^4^I_13/2_→^4^I_15/2_ transition, its magnetic dipole transition rate can be calculated from following Eq. (S4) to be 82.83 s^-1^.

$A_{4I13\to4I15/2}^{MD}=\frac{16\pi^{4}e^{2}n^{3}}{3h(2J+1)m^{2}c^{2}}\left| \left\langle\left( S,L \right)J\left| L+2S \right|\left( S',L' \right)J' \right\rangle\right|^{2}$ (S4)

where $\left\langle\left( S,L \right)J\left| L+2S \right|\left( S',L' \right)J' \right\rangle$ is reduced matrix element for the magnetic dipole transition. Therefore, the real Judd-Ofelt parameters can be derived from the formula below.

$\Omega_{\lambda}=\frac{\frac{1}{\tau_{ex}} -A_{4I13\to4I15/2}^{MD}}{A_{4I13\to4I15/2}^{'ED}}{\Omega'}_{\lambda}$ (S5)

Here, $A_{4I13\to4I15/2}^{'ED}$ is the relative radiative electric-dipole-allowed transition rate for ^4^I_13/2_→^4^I_15/2_ transition and can be calculated directly from Eq. (S6) below.

$A_{4I13\to4I15/2}^{'ED}=\frac{64\pi^{4}e^{2}v^{3}n\left( n^{2}+2 \right)^{2}}{27h\left( 2J+1 \right)}\sum_{\lambda=2, 4, 6} {\Omega_{\lambda}^{'}\left| \left\langle\left( S,L \right)J\left| U^{\lambda} \right|\left( S',L' \right)J' \right\rangle\right|}^{2}$ (S6)

From the above calculation, the real Judd-Ofelt parameters are confirmed to be $\Omega_{2}=$3.65×10^-19^ cm^2^, $\Omega_{4}=$ 4.31×10^-20^ cm^2^, and $\Omega_{6}=$ 2.09×10^-21^ cm^2^. Once the Judd-Ofelt parameters are confirmed the radiative transition rate for all transitions between 4f levels of Er^3+^ in NaY(WO_4_)_2_ can be calculated using Eq. (S4) for magnetic dipole transition and Eq. (S6) for electric dipole transition, and the calculation results are listed in Table 1 as 4^th^ column. These radiative transition rates will be used in calculating the quantum cutting efficiency.

**<Fig. S2>**


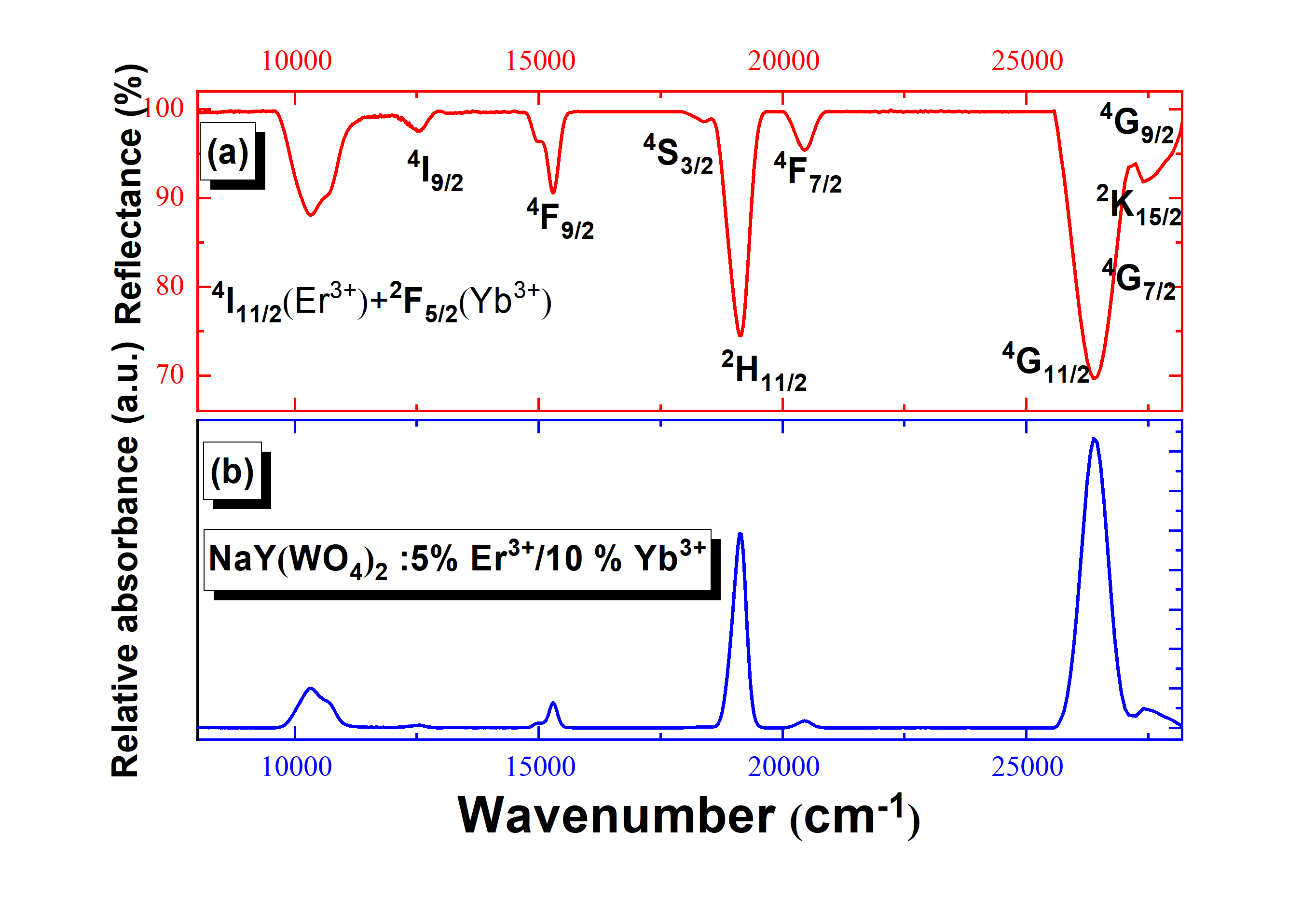


Fig. S2 Defuse-reflection (a) and relative absorption (b) spectra of NaY(WO_4_)_2_ :5 mol% Er^3+^/10 mol% Yb^3+^

**<Fig. S3>**


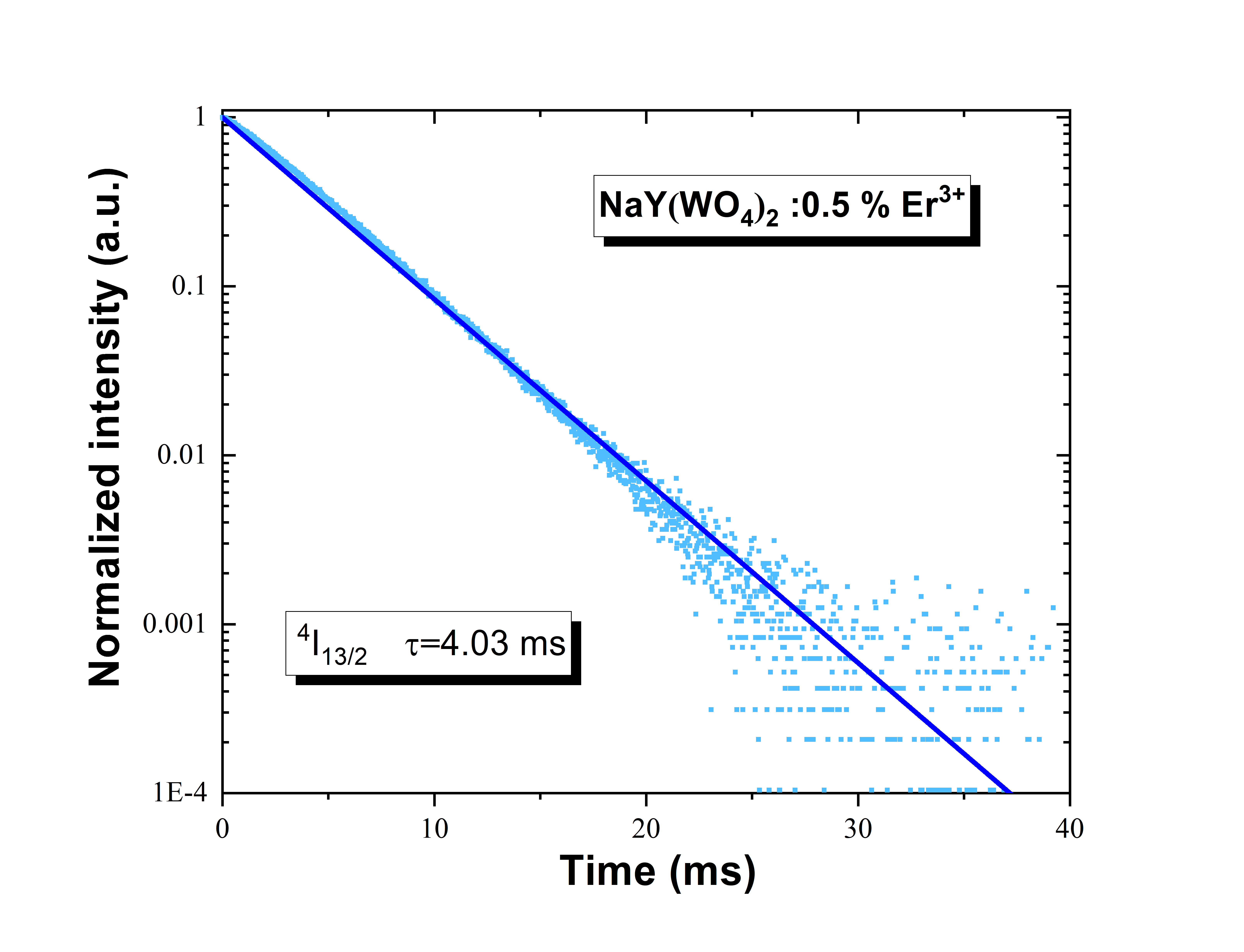


Fig. S3 Fluorescence decay for ^4^I_13/2_→^4^I_15/2_ transition of Er^3+^ in NaY(WO_4_)_2_ :0.5 mol% Er^3+^ phosphor. The solid curve is the fitting result by using single exponential function.

**<Fig. S4>**


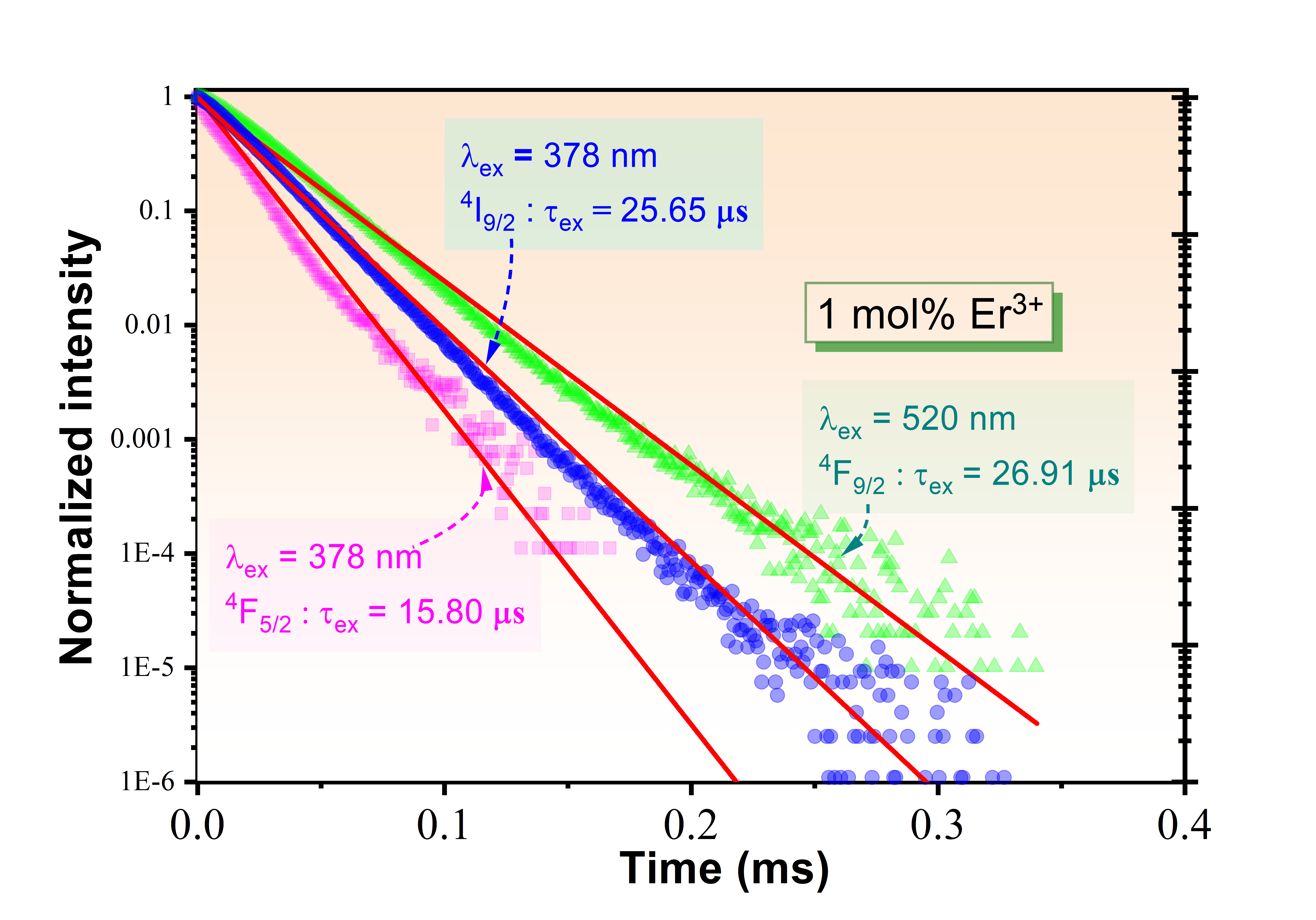


Fig. S4 Fluorescence decays for ^4^F_5/2_ (under 378 nm excitation), ^4^F_9/2_ (under 520 nm excitation), and ^4^I_9/2_ (under 378 nm excitation)

**<Fig. S5>**


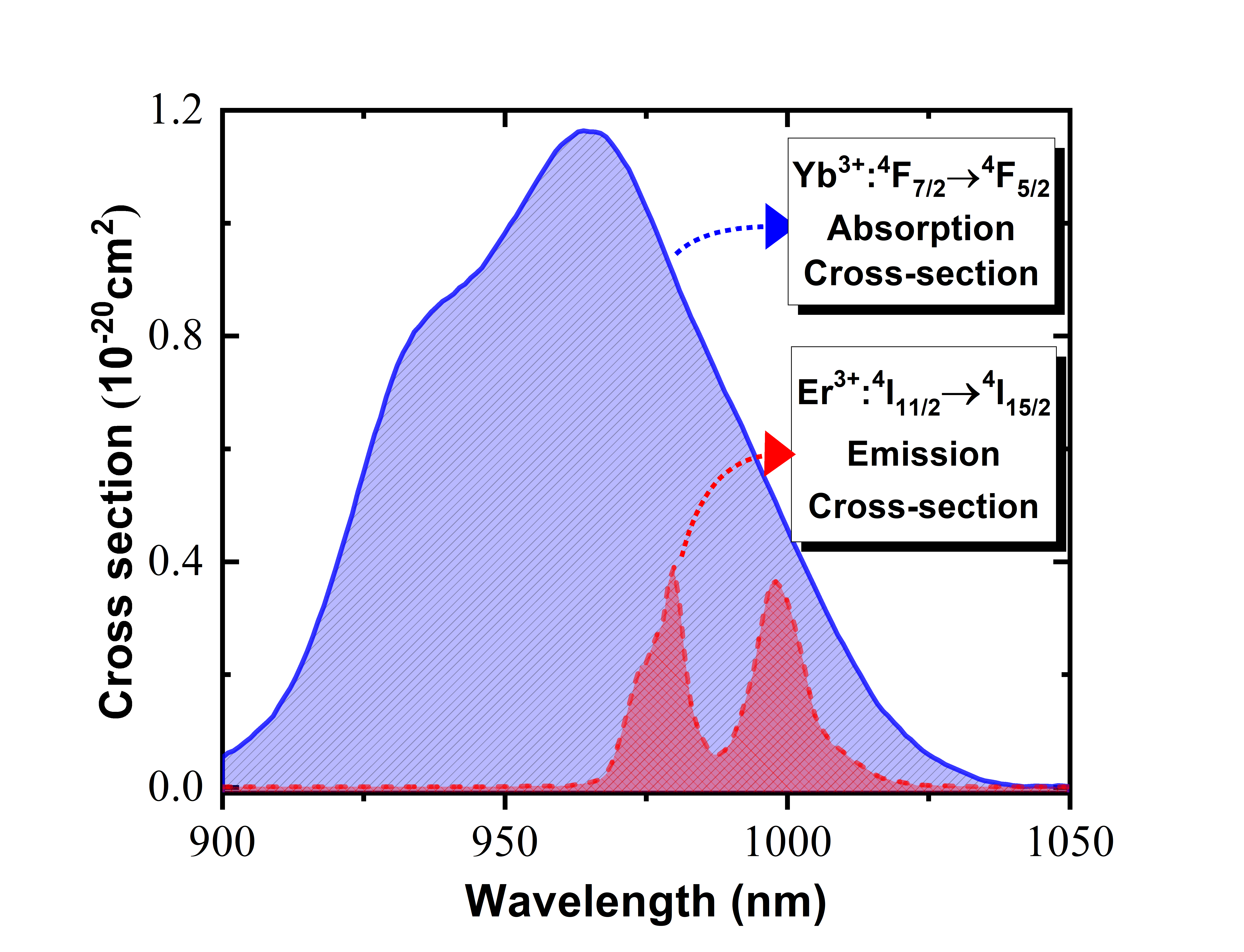


Fig. S5 Dashed curve: emission cross-section of ^4^I_11/2_→^4^I_15/2_ transition of Er^3+^ in NaY(WO_4_)_2_; Solid curve: absorption cross-section of ^2^F_7/2_→^2^F_5/2_ transition of Yb^3+^ in NaY(WO_4_)_2._,

**<Fig. S6>**


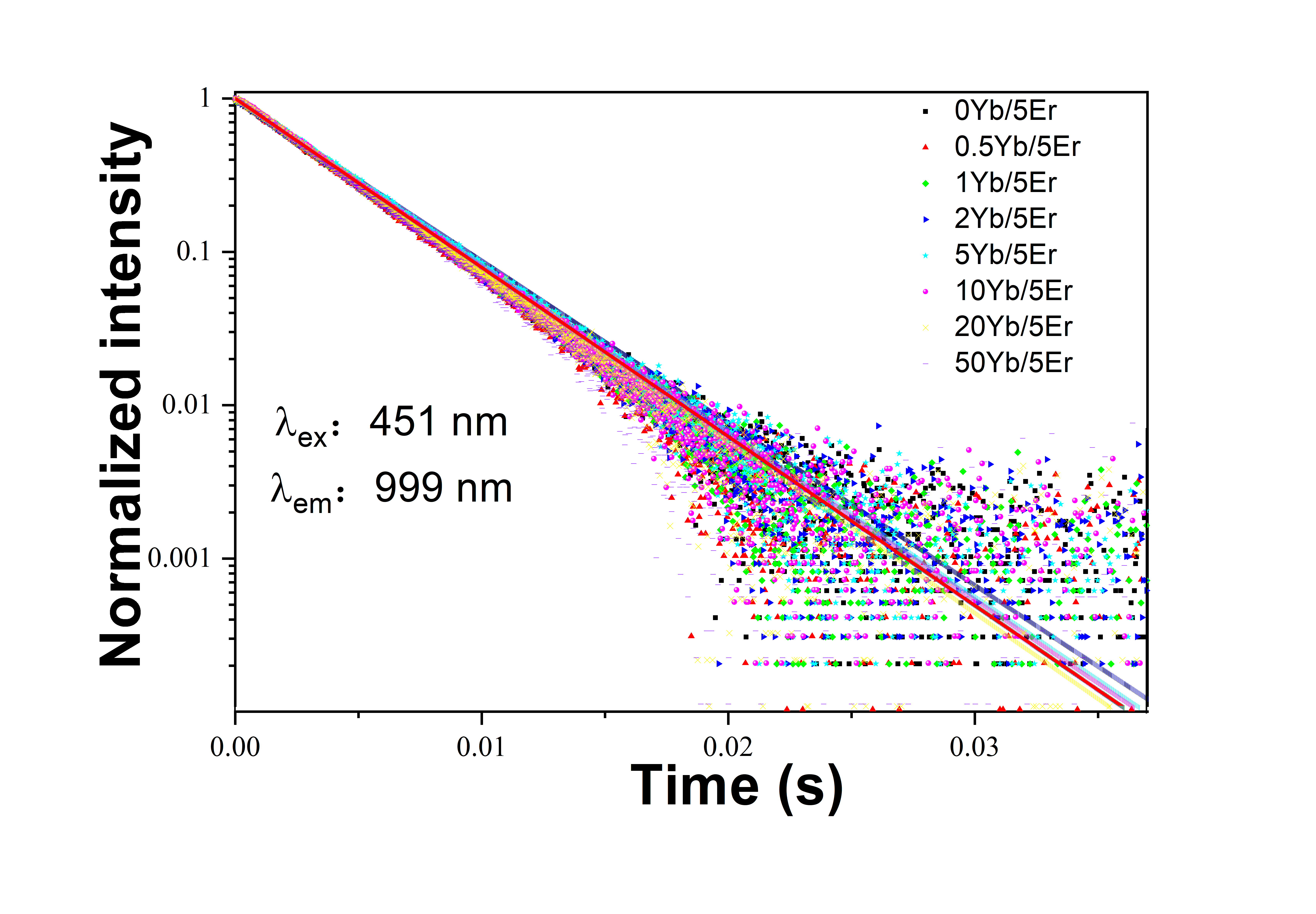


Fig. S6 Fluorescence decay for ^2^F_5/2_→^2^F_7/2_ transition of Yb^3+^ in NaY(WO_4_)_2_ :5 mol% Er^3+^/x mol%Yb^3+^ (x=0, 0.5, 1, 2, 5, 10, 20, 50) phosphors.

**<Fig. S7>**


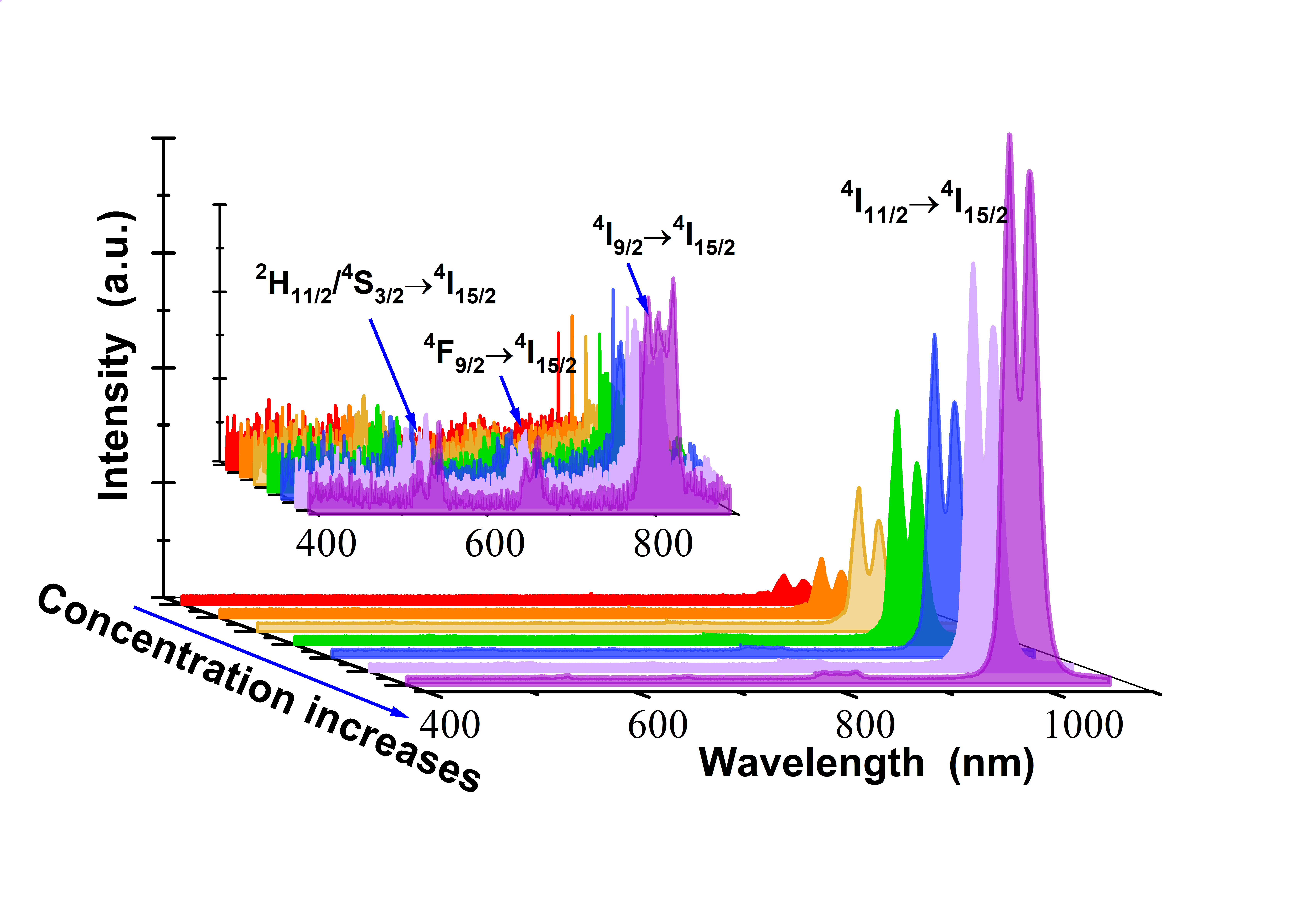


Fig. S7 Up-conversion emission spectra of NaY(WO_4_)_2_: *x* mol% Er^3+^ (*x* = 0.5, 1, 2, 5, 10, 20, 50) under 1550 nm excitation

**<Fig. S8>**


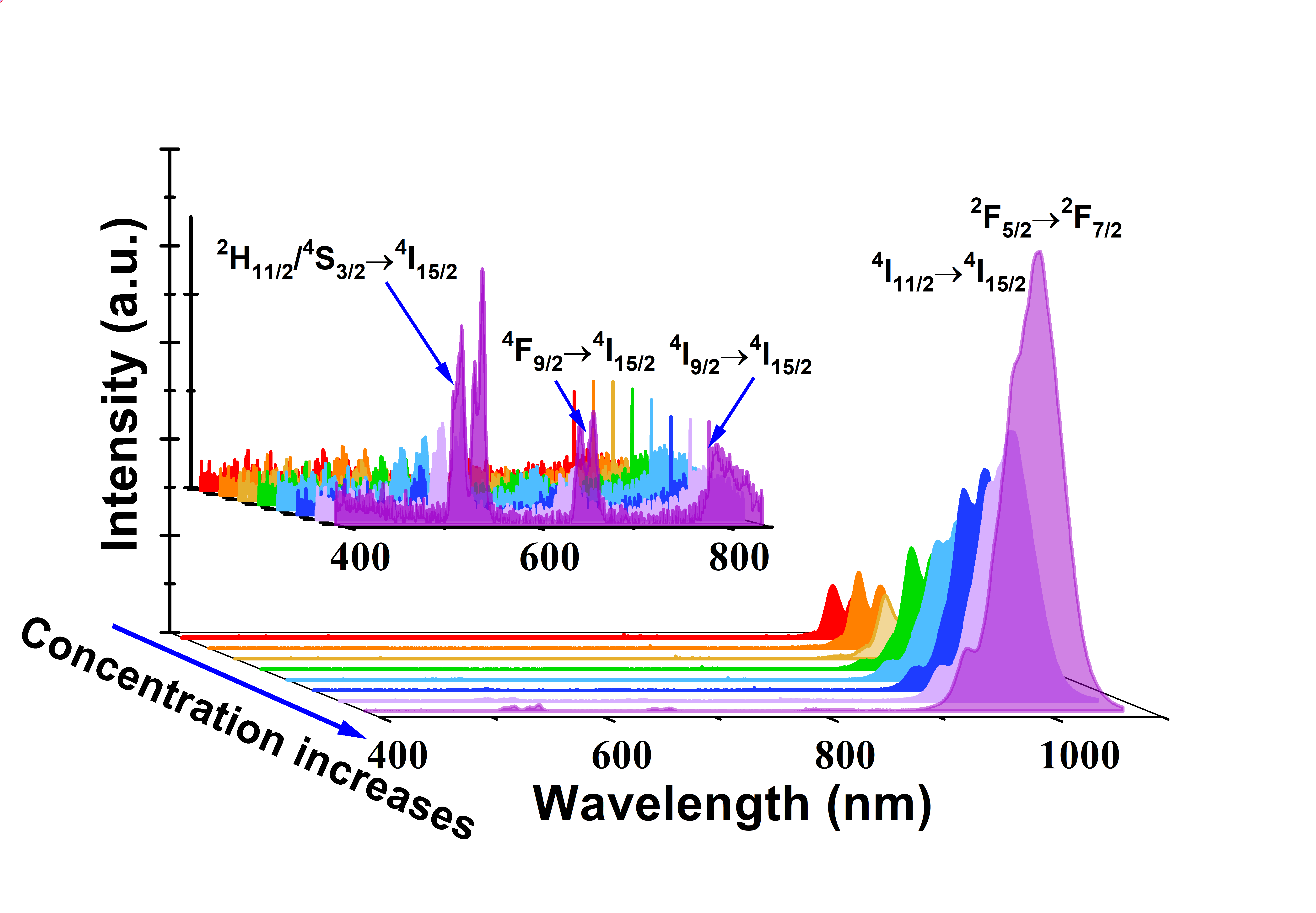


Fig. S8 Up-conversion emission spectra of NaY(WO_4_)_2_: 5 mol% Er^3+^/*x* mol% Yb^3+^ (*x* = 0, 0.5, 1, 2, 5, 10, 20, 50) under 1550 nm excitation;

**<Fig. S9>**


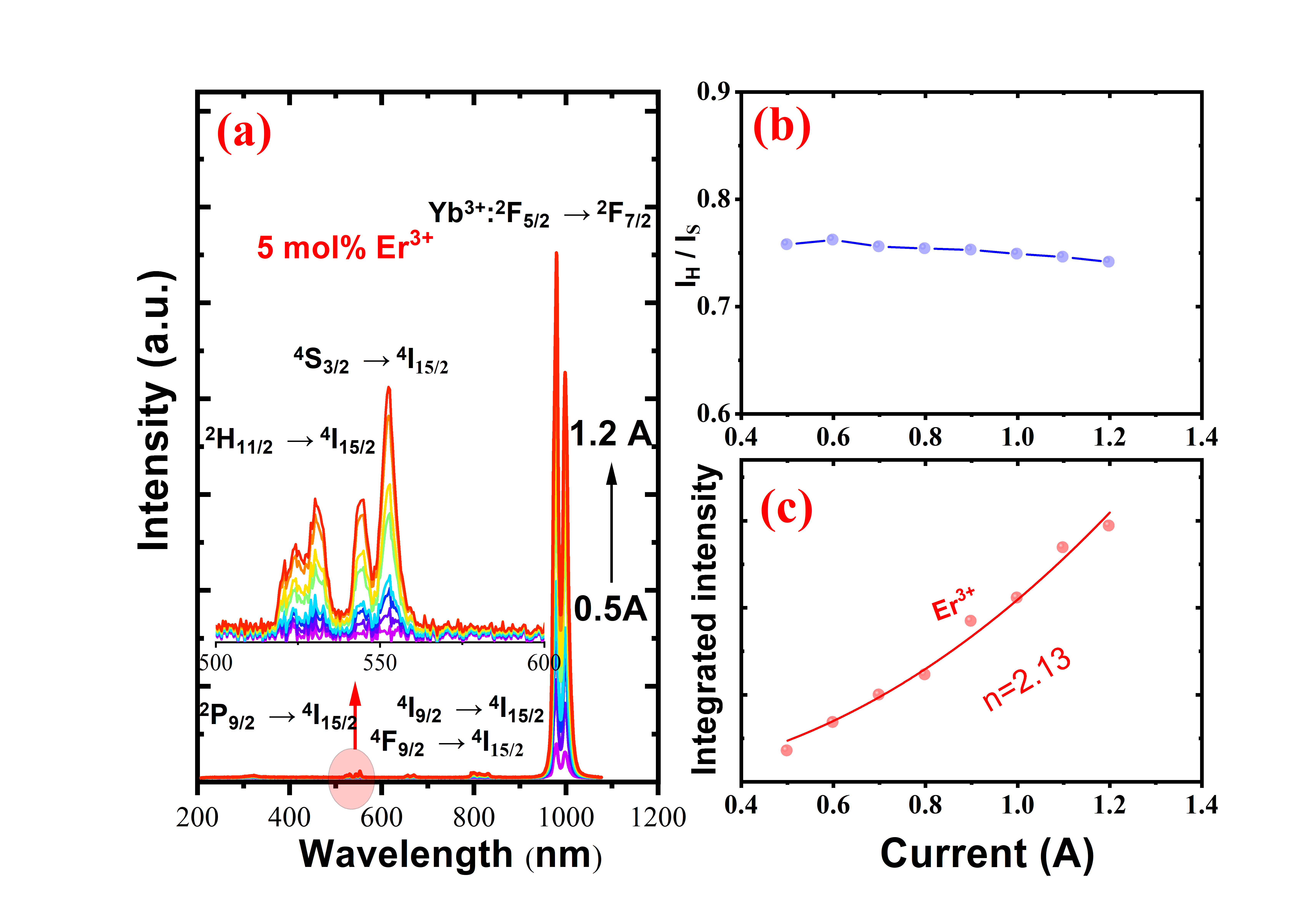


Fig. S9 (a) Upconversion emission spectra for 5 mol% Er^3+^ doped NaY(WO_4_)_2_ phosphor excited at varied excitation power under 1550 nm laser, (b) relation between fluorescence intensity ratio of ^2^H_11/2_→^4^I_15/2_ to ^4^S_3/2_→^4^I_15/2_ and the working current, (c) dependence of the integrated upconversion intensity for ^4^I_11/2_→^4^I_15/2_ emission on the working current

**<Fig. S10>**


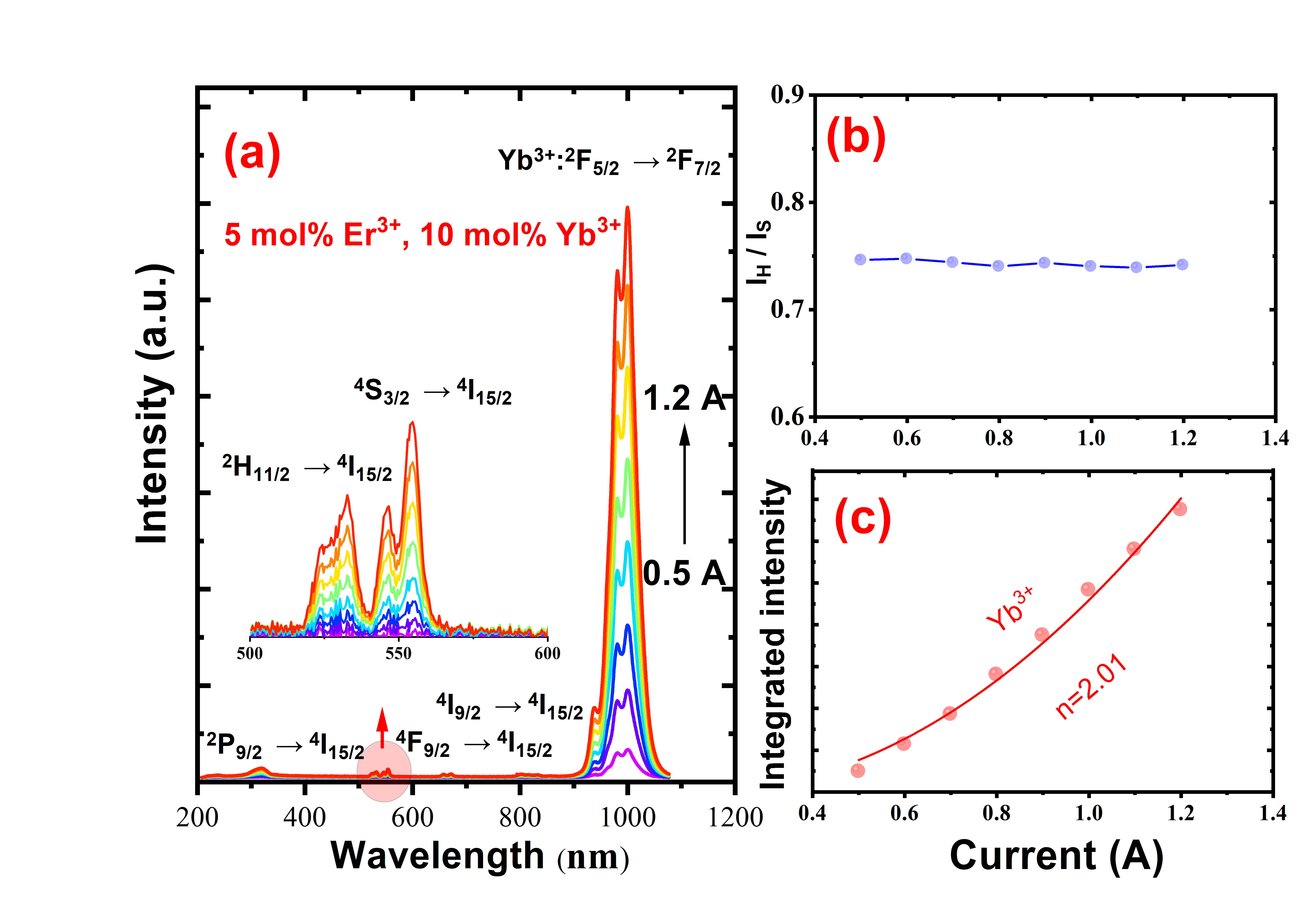


Fig. S10 (a) Upconversion emission spectra for 5 mol% Er^3+^/10 mol% Yb^3+^ doped NaY(WO_4_)_2_ phosphor excited at varied excitation power under 1550 nm laser, (b) relation between fluorescence intensity ratio of ^2^H_11/2_→^4^I_15/2_ to ^4^S_3/2_→^4^I_15/2_ and the working current, (c)dependence of the integrated upconversion intensity for ^2^F_5/2_→^2^F_7/2_ emission on the working current

**<Fig. S11>**


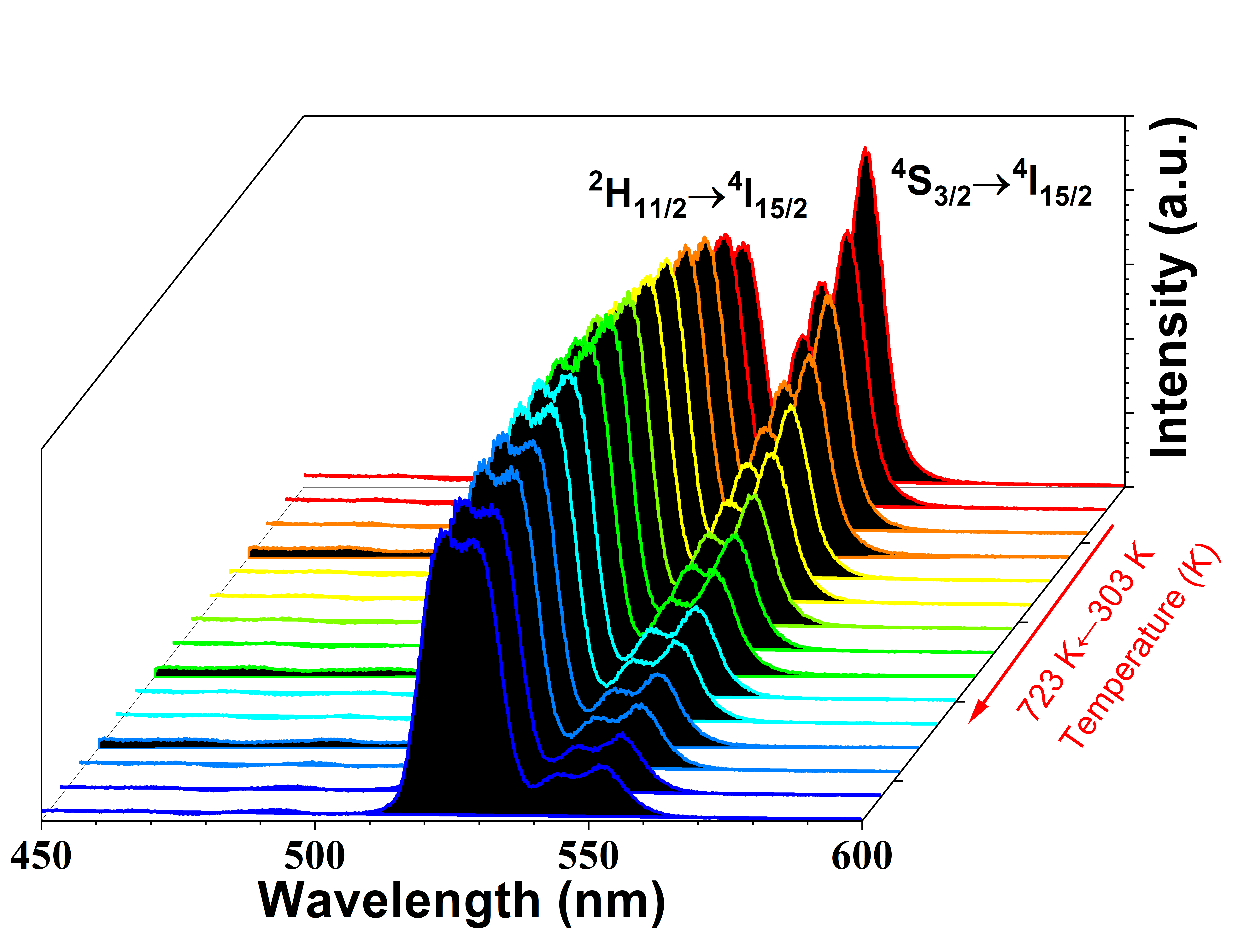


Fig. S11 Green emission spectra of 5 mol% Er^3+^ and 10 mol% Yb^3+^ co-doped NaY(WO_4_)_2_ phosphor were measured at different temperatures from 303 K to 723 K under 378 nm excitation

**<Table S1>**

Table S1 Relative experimental and theoretical oscillator strengths for some transitions of Er^3+^, error, relative and absolute J-O parameters

| Energy  level | Relative experimental oscillator strengths(10^-8^) | Relative theoretical oscillator strengths(10^-8^) |
| --- | --- | --- |
| ^4^I_9/2_ | 0.33 | 0.4 |
| ^4^F_9/2_ | 1.87 | 1.81 |
| ^4^S_3/2_ | 0.11 | 0.04 |
| ^2^H_11/2_ | 19.6 | 27.07 |
| ^4^F_7/2_ | 0.69 | 0.76 |
| ^4^G_11/2_ | 55.86 | 47.8 |
| Error=6.345  Relative J-O parameters: *Ω_2_'* = 1.44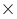10^-21^ cm^2^; *Ω_4_'* =1.70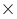10^-22^ cm^2^; *Ω_6_'* = 8.23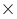10^-24^ cm^2^  Absolute J-O parameters: *Ω_2_'* = 3.65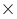10^-19^ cm^2^; *Ω_4_*' =4.31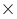10^-20^ cm^2^; *Ω_6_'* = 2.09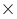10^-21^ cm^2^ | | |

**<Table S2>**

Table S2 Quantum cutting efficiencies for all the samples

| NaY(WO_4_)_2_: 5 mol% Er^3+^ doped | Quantum cutting efficiency (%) |
| --- | --- |
| 0 mol%Yb^3+^ | 0 |
| 0.5 mol%Yb^3+^ | 53.51 |
| 1 mol%Yb^3+^ | 56.00 |
| 2 mol%Yb^3+^ | 64.64 |
| 5 mol%Yb^3+^ | 90.07 |
| 10 mol%Yb^3+^ | 112.45 |
| 20 mol%Yb^3+^ | 138.72 |
| 50 mol%Yb^3+^ | 173.57 |
